# Transcription profiling of feline mammary carcinomas and derived cell lines reveals biomarkers and drug targets associated with metabolic and cell cycle pathways

**DOI:** 10.1038/s41598-022-20874-5

**Published:** 2022-10-11

**Authors:** José Luis Granados-Soler, Leila Taher, Julia Beck, Kirsten Bornemann-Kolatzki, Bertram Brenig, Verena Nerschbach, Fernando Ferreira, Johannes Junginger, Marion Hewicker-Trautwein, Hugo Murua Escobar, Ingo Nolte

**Affiliations:** 1 School of Veterinary Science, The University of Queensland, Gatton, Australia; 2 Institute of Biomedical Informatics, Graz University of Technology, Graz, Austria; 3 Institute for Biostatistics and Informatics in Medicine and Ageing Research, Rostock University Medical Center, Rostock, Germany; 4 Chronix Biomedical, Göttingen, Germany; 5 Institute of Veterinary Medicine, University of Göttingen, Göttingen, Germany; 6 Small Animal Clinic, University of Veterinary Medicine Hannover Foundation, Hannover, Germany; 7 Faculty of Veterinary Medicine, CIISA – Centre for Interdisciplinary Research in Animal Health, University of Lisbon, Lisbon, Portugal; 8 Associate Laboratory for Animal and Veterinary Sciences (AL4AnimalS), Lisbon, Portugal; 9 Department of Pathology, University of Veterinary Medicine Hannover Foundation, Hannover, Germany; 10 Haematology, Oncology and Palliative Medicine, Clinic III, University of Rostock, Rostock, Germany

**Keywords:** Cancer, Molecular biology, Biomarkers, Oncology

## Abstract

The molecular heterogeneity of feline mammary carcinomas (FMCs) represents a prognostic and therapeutic challenge. RNA-Seq-based comparative transcriptomic profiling serves to identify recurrent and exclusive differentially expressed genes (DEGs) across sample types and molecular subtypes. Using mass-parallel RNA-Seq, we identified DEGs and performed comparative function-based analysis across 15 tumours (four basal-like triple-negative [TN], eight normal-like TN, and three luminal B *f*HER2 negative [LB *f*HER2−]), two cell lines (CL, TiHo-0906, and TiHo-1403) isolated from the primary tumours (LB *f*HER2−) of two cats included in this study, and 13 healthy mammary tissue controls. DEGs in tumours were predominantly upregulated; dysregulation of CLs transcriptome was more extensive, including mostly downregulated genes. Cell-cycle and metabolic-related DEGs were upregulated in both tumours and CLs, including therapeutically-targetable cell cycle regulators (e.g. *CCNB1*, *CCNB2*, *CDK1*, *CDK4*, *GTSE1*, *MCM4*, and *MCM5)*, metabolic-related genes (e.g. *FADS2* and *SLC16A3*), heat-shock proteins (e.g. *HSPH1, HSP90B1*, and *HSPA5*), genes controlling centrosome disjunction (e.g. *RACGAP1* and *NEK2*), and collagen molecules (e.g. *COL2A1*). DEGs specifically upregulated in basal-like TN tumours were involved in antigen processing and presentation, in normal-like TN tumours encoded G protein-coupled receptors (GPCRs), and in LB *f*HER2− tumours were associated with lysosomes, phagosomes, and endosomes formation. Downregulated DEGs in CLs were associated with structural and signalling cell surface components. Hence, our results suggest that upregulation of genes enhancing proliferation and metabolism is a common feature among FMCs and derived CLs. In contrast, the dissimilarities observed in dysregulation of membrane components highlight CLs’ disconnection with the tumour microenvironment. Furthermore, recurrent and exclusive DEGs associated with dysregulated pathways might be useful for the development of prognostically and therapeutically-relevant targeted panels.

## Introduction

Mammary carcinomas in cats are predominantly triple-negative (Oestrogen Receptor negative [ER-], Progesterone Receptor negative [PR-], and feline orthologous of the human epidermal growth factor receptor 2 negative [*f*HER2−]) resembling aggressive human breast cancer (HBC) subtypes^1–5^. As observed in human breast oncology^6–9^, feline mammary carcinomas (FMCs) represent a heterogeneous group of diseases with distinctive molecular traits influencing therapeutic response and survival intervals^2,5,10^. Accordingly, a detailed characterisation of FMCs molecular subtypes and derived in vitro model’s gene dysregulation is pivotal for the development and testing of molecularly-targeted therapies^11^.

The heterogeneity, biological behaviour, and prognostic differences among FMCs have been realised through histopathology, immunohistochemical profiling and more recently by the emergence of in-depth nucleic acid analyses^2,5,10,12–15^. Molecular subtypes described include luminal A (LA), luminal B *f*HER2 negative (LB *f*HER2−), LB *f*HER2 positive (LB *f*HER2+), *f*HER2 positive [*f*HER2+], basal-like triple-negative (TN), and normal-like TN. Moreover, FMCs and HBCs molecular subtypes are intrinsically heterogeneous^6–9,16–18^. Thus, characterising gene dysregulation through deeper molecular analyses is imperative to identify common and specific molecular markers and therapeutic targets across molecular subtypes.

Immortalised CLs are useful to study specific features of the neoplastic process and to test novel treatment modalities under controlled conditions. Despite establishment and characterisation of FMC-derived CLs^19–26^, their actual potential to model distinctive aspects of the neoplastic transformation and suitability for the testing of specific therapeutic alternatives is yet to be elucidated.

High-resolution transcriptome sequencing serves to detect up-, or downregulated DEGs in neoplastic lesions and derived CLs in comparison to healthy tissues. Furthermore, investigating DEGs using available online resources for function-based analysis (e.g. Gene Ontology and DAVID functional analysis) serves to provide biological insight into molecular mechanisms behind cancer, as DEGs are categorised into gene sets based on cellular location, shared biological function, and/or involvement in molecular pathways. Comparing results of function-based DEGs analysis across different molecular subtypes has the potential of revealing specific features characterising the neoplastic dysregulation and possible prognostic and therapeutic implications, on the other hand, a comparison across CLs and tumour samples, helps to reveal changes associated with cell seeding and subculturing favouring clonal selection.

Thus, this study aimed to identify recurrent and exclusive DEGs across 15 tumours representing three immunohistochemically defined molecular subtypes (basal-like TN, normal-like TN, and LB *f*HER2−) and two CLs derived from LB *f*HER2− tumours in comparison with healthy mammary tissues including control specimens derived from the same individuals. To disclose possible similarities and differences among primary lesions and established cellular models, we analysed recurrent and exclusive DEG sets for enrichment in specific cellular components, biological processes, molecular functions, and signalling pathways.

## Results

### Animals and samples characterisation

We generated and analysed RNA-seq libraries of 15 selected primary tumours (eight formalin-fixed paraffin-embedded [FFPE] and seven fresh-frozen tissue [FT] samples), three samples representing two cell lines (CLs) at different passages (TiHo-1403 passage 10, and TiHo-0906^26^ passage 7 and 77), and 13 healthy mammary tissue controls (FFPE = 12 and FT = 1). Seven of the primary tumour samples had a healthy mammary tissue control counterpart obtained from the same animal; CLs were isolated from the primary tumours of two animals (Fig. S1). Characteristics of patients and tumours included in this study are summarised in Table S1. After immunohistochemical evaluation of ER, PR, *f*HER2, CK5/6, and Ki-67 and validation of *f*HER2 amplification using mass-parallel sequencing^10^, tumours were classified following the St. Gallen guidelines as basal-like TN (n = 4, 26.67%), normal-like TN (n = 8, 53.33%), and LB *f*HER2− (n = 3, 20%). CL samples were derived from LB *f*HER2− tumours. TiHo-1403 displayed 26 h doubling time at passage 10 and was able to proliferate until passage 33. TiHo-0906 cellular morphology and proliferation rate during prolonged subculturing have been previously documented^26^.

Animals included were followed up for two years. After censoring and survival analysis, cats with basal-like TN tumours displayed the poorest survival (n = 4; disease-free interval [DFI]: 3.35 ± 1.71 months, and cancer-specific overall survival [CSS]: 3.73 ± 1.67 months), followed by normal-like TN (n = 7; DFI: 7.22 ± 4.87 months, and CSS: 9.34 ± 4.28 months), and LB *f*HER2− (n = 2; DFI: 12 ± 5.9, and CSS: 14.3 ± 4.2 months), as previously reported^2,5,10^. Additional outcome information of included cases is provided in Table S2.

### The transcriptome of CLs is more dysregulated and includes more downregulated genes compared to tumours

Importantly, gene expression profiles were more similar among samples of the same type (i.e., CL, and FT and FFPE tumours) than to molecular subtype (i.e., LA, LB *f*HER2−, normal-like and basal-like TN, Fig. S1). Based on their expression profiles, CLs and FT samples formed a cluster that was clearly separated from the cluster formed by FFPE samples. Both clusters included a mixture of controls and neoplastic samples. Paired samples (control/neoplastic and cell line/original tumour) were separated into different subclusters.

**Figure 1:**
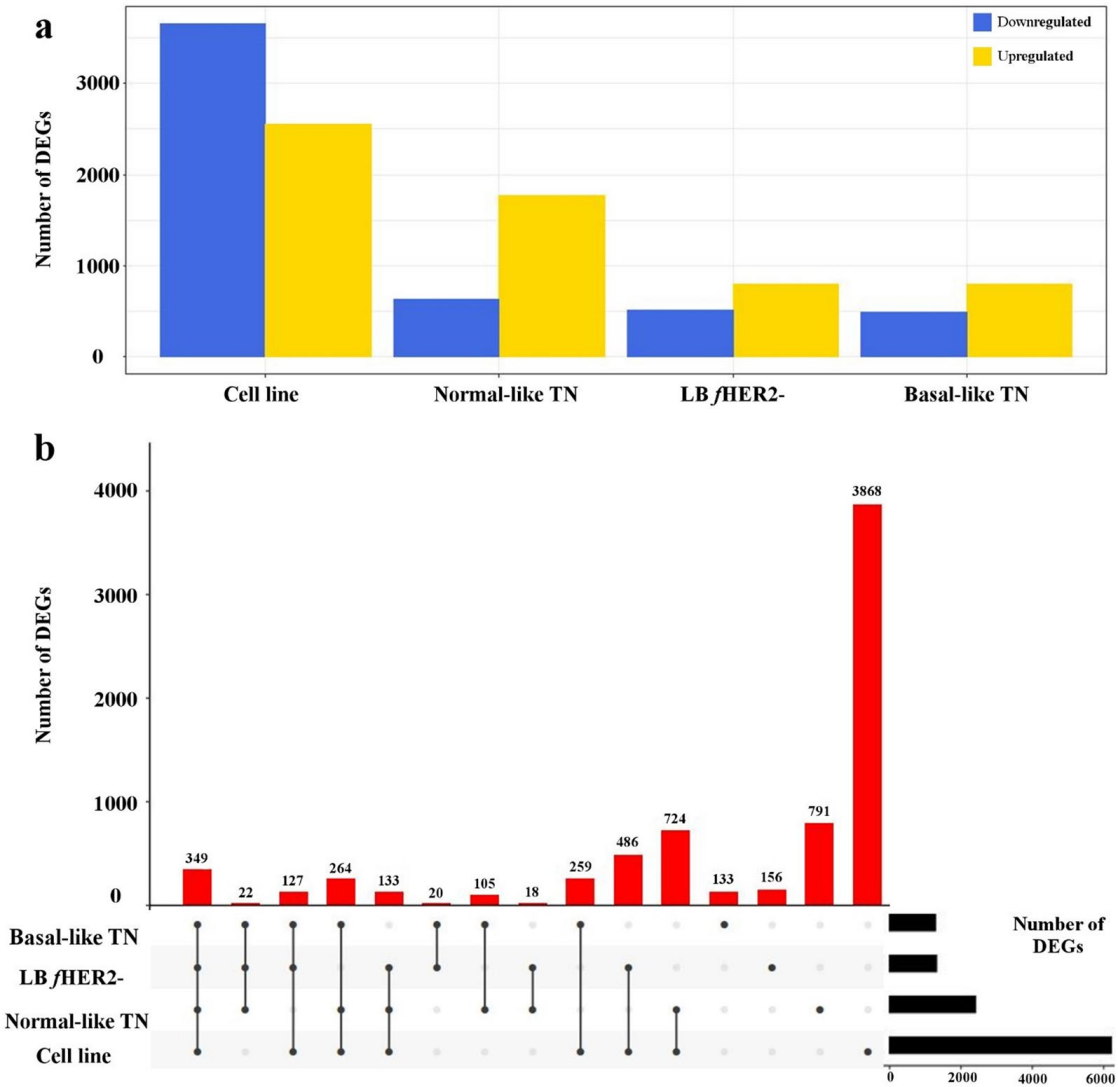
Differentially expressed genes (DEGs) relative to control samples for the four sample groups considered in this study. (**a**) Number of up- and down-regulated DEGs. (**b**) UpSet plot^27^ showing disjoint intersections of DEGs in different comparisons. LB *f*HER2-, luminal B *f*HER2 negative tumours; and *TN* triple-negative tumours. A total of 349 DEGs were recurrently dysregulated across all four sample groups.

Independent differential gene expression analysis between each neoplastic sample group (i.e., CLs, LB *f*HER2− tumours, normal-like TN tumours, and basal-like TN tumours) and control samples identified a total of 7,454 DEGs out of 18,504 detected protein-coding genes (adjusted *P* value < 0.05 and log_2_ fold-changes ≥ 2 or ≤ −2; Methods). Among all sample groups studied, CLs displayed the highest number of DEGs (n = 6210), with 62% of them being exclusively differentially expressed in this sample group. Among primary neoplastic samples, normal-like TN tumours had the highest number of DEGs (n = 2406), followed by LB *f*HER2− (n = 1310), and basal-like TN (n = 1279). DEGs exclusive to tumour samples represented 10.39–32.87% of DEGs, depending on the subtype (Fig. 1a). While most DEGs in tumours were upregulated, DEGs in CLs were predominantly downregulated (Fig. 1a). Furthermore, CLs featured the highest number of extremely dysregulated genes (log_2_ fold-changes ≥ 5 and ≤ −5), representing 12.25% of DEGs in this sample group. Indeed, most DEGs (97.25–99.42%, depending on the subtype) in tumours were only moderately dysregulated (log_2_ fold-changes ≥ 2 but ≤ 5, and ≤ -2 but ≥ −5). Only 349 DEGs (~ 5% of all DEGs, Fig. 1b) were recurrently dysregulated across all sample groups. Specifically, 203 DEGs were consistently upregulated and 137 were downregulated across all sample groups (Table S3), while the additional nine genes were dysregulated in different directions depending on the sample group.

For each sample group, we then identified ten “top DEGs” (Table S4; Methods). *RACGAP1* was among the top DEGs in all sample groups. *NEK2* was among the top DEGs in triple-negative subtypes and was upregulated across all samples. Furthermore, top upregulated genes across different tumour subtypes and CLs included several proliferation-related and metabolic-related therapeutic targets in cancer. On the other hand, top downregulated genes included ATP-binding cassette (ABC) transporters, chemotherapy resistance-associated genes, metabolic-related genes, cell adhesion molecules, and membrane receptors (Table S4).

### Recurrently upregulated genes across all sample groups were associated with cell cycle and metabolic-related pathways

DEGs that were recurrently upregulated across all sample groups (n = 203) were associated with pathways related to cell cycle and metabolic networks (Table S5 Methods). Consistently, similar pathways were also enriched among the DEGs detected in each sample group (Tables S6–9). Besides the upregulation of cell cycle and metabolic-related pathways observed for all sample groups, several other pathways supporting replication, transcription, translation, and protein processing were specifically associated with the DEGs upregulated in CLs (Table S9).

In agreement with the pathway enrichment analysis, gene ontology (GO) analysis revealed that DEGs that were recurrently upregulated across all sample groups represented protein complexes relevant during DNA replication and cellular division (e.g., DNA highly conserved mini-chromosome maintenance [MCM], and Ndc80 complexes), or were involved in movement elicitation (e.g. kinesin complexes) and cellular parts pivotal during cytokinesis (e.g. kinetochore, microtubule cytoskeleton, mitotic spindle, and midbody). Accordingly, GO terms representing molecular functions and biological processes were mostly related to cell cycle, motor activity, and cytoskeleton reorganisation during mitosis (Fig. S2).

### Upregulated genes through all tumour subtypes and cell lines were associated with three cell cycle-related pathways

DEGs that were recurrently upregulated across all sample groups (n = 137) were associated with three interconnected pathways governing key events during cell cycle progression and control: DNA replication, cell cycle, and p53 signalling (Table S5). These pathways were also enriched among the DEGs detected in each sample group (Tables S6–9). Recurrently upregulated DEGs across all sample groups associated with enhanced proliferation included core cell cycle regulators (e.g., *CCNB1*, *CCNB2*, *CDK1*, and *CDK4*) and genes promoting DNA synthesis, and/or repair (e.g., *MCM4* and *MCM5*). In addition to these genes, the different sample groups featured their own specific cell cycle regulators. For example, the histone deacetylase gene *HDAC1* was upregulated across all sample groups except for LB *f*HER2− tumours, and the histone deacetylase gene *HDAC2* was only upregulated in CLs (Tables S6–9).

### The concurrent up-regulation of TP53 and its downstream effectors related to proliferation suggests a loss of p53 tumour suppressor activity in tumours and derived cell lines

**Figure 2:**
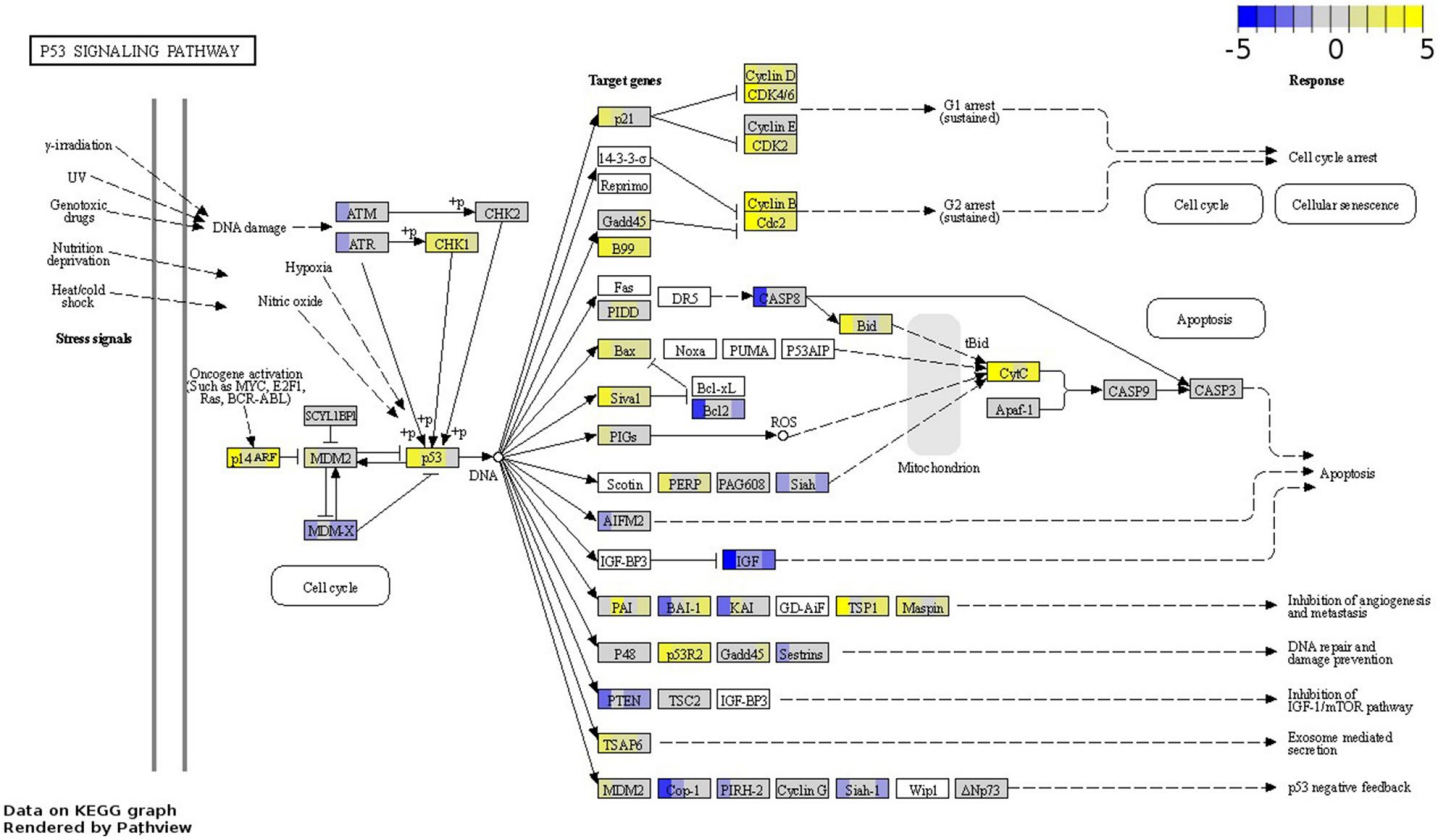
Differentially expressed genes associated with the p53 signalling pathway (fca04115) across all sample groups. The boxes illustrating DEGs are uniformly divided by the number of sample groups; representing from left to right: CLs, LB *f*HER2− tumours, normal-like TN tumours, and basal-like TN tumours. Gene log_2_ fold-change relative to controls is indicated in yellow (upregulated) or blue (downregulated), white boxes represent genes not detected. Data on KEGG graph rendered by “Pathview” (https://pathview.uncc.edu/).

The p53 pathway is implicated in the cellular homeostatic mechanisms controlling cell proliferation in response to a variety of intrinsic and extrinsic stress signals and is commonly disrupted in cancer^28–30^. Interestingly, in all sample groups, *TP53* and various p53 target genes were upregulated. Typically, p53 targets (e.g., *CDKN1A*, *GTSE1, GADD45A*) interact with cyclins and cyclin-dependent kinases involved in cell cycle progression to inhibit their expression^31^. But surprisingly, the cyclins and cyclin-dependent kinases *CCNB1*, *CCNB2*, *CDK1*, and *CDK4* were upregulated in all sample groups (Table S3). Moreover, also the p53 activation mediator *CHEK1*, the p53 downstream effector *RRM2*—involved in DNA repair—, and *GTSE1*—which represses p53’s ability to induce apoptosis (Table S5 and Fig. 2)^32–35^—were upregulated in all sample groups. On the other hand, kinases favouring p53 activation in response to stress cues, such as *ATM* and *ATR* —which signals are integrated by *CHEK1*— were only downregulated in CLs. Overall, a stronger dysregulation of the p53 pathway was observed in CLs when compared to tumours (Tables S6–9). Notably, mechanisms underlying oncogene-mediated activation of p53 appear to be more active in CLs and basal-like tumours, as evidenced by the upregulation in both groups of *CDKN2A—*which indirectly activates p53 by repressing *MDM2*^36,37^. However, the main negative feedback of *TP53* (*MDM2*) was upregulated in CLs, while *MDM4* which inhibits p53 by binding its transcriptional activation domain^29^, was downregulated in this group. Finally, despite upregulation of TP53 and p53 target genes, downstream related proliferation signals remained active across all sample groups.

### PI3K/Akt/mTOR pathway activation was downregulated in CLs while AKT-related cell proliferation and survival remained active across all sample groups except for basal-like tumours

**Figure 3:**
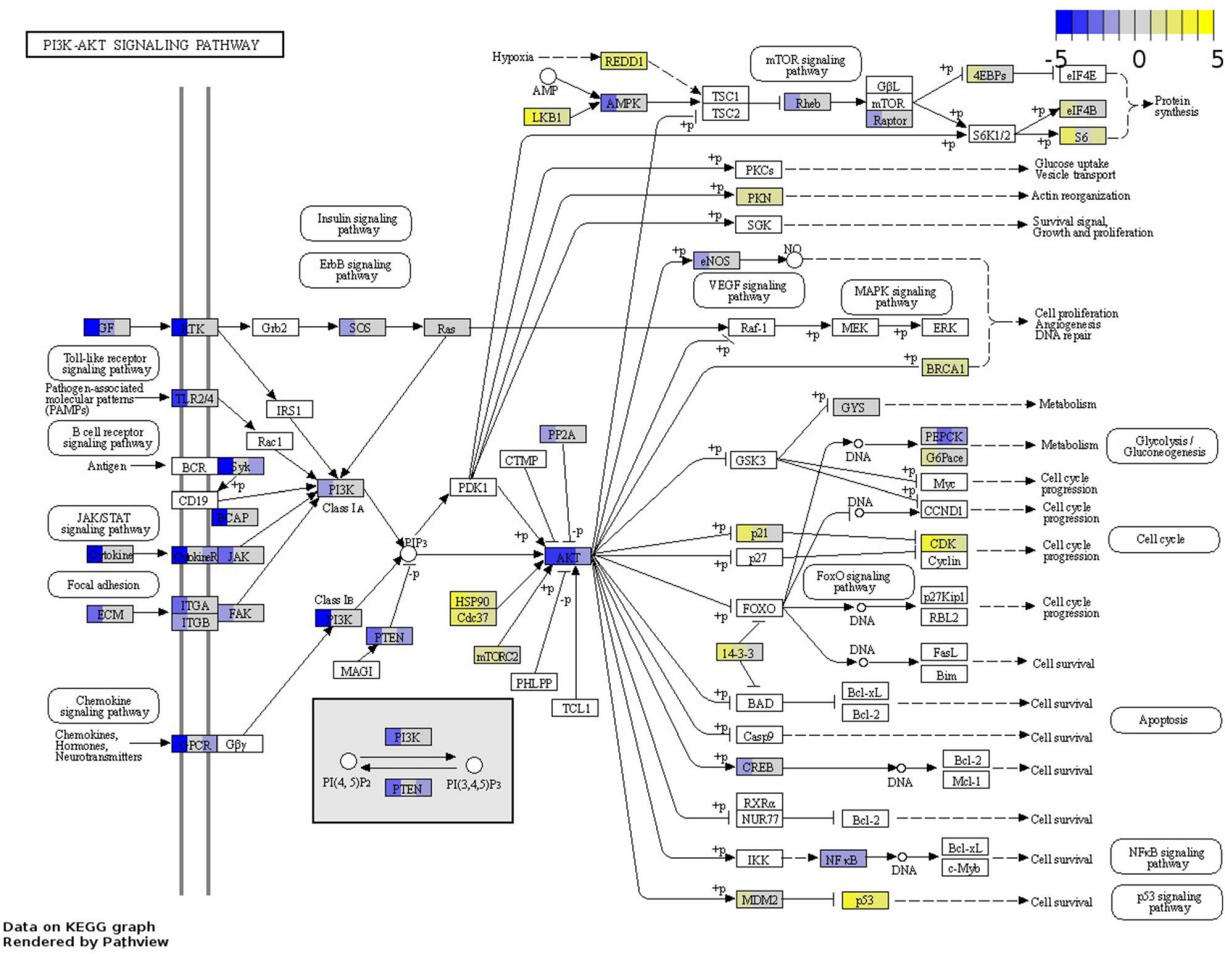
Differentially expressed genes enriching the PI3K-AKT signalling pathway (fca04151) in CLs, LB *f*HER2-, and normal-like TN tumours. The boxes illustrating DEGs are uniformly divided by the number of sample groups; representing from left to right: CLs, LB *f*HER2- tumours, and Normal-like TN tumours. Gene log_2_ fold change relative to controls is indicated in yellow (upregulated) or blue (downregulated), white boxes represent genes not detected. Data on KEGG graph rendered by “Pathview” (https://pathview.uncc.edu/).

The PI3K/Akt/mTOR pathway is implicated in the regulation of cell growth, proliferation, metabolism, and motility^38^, and several of its components are commonly activated in cancer^39^. And indeed, the DEGs in each of the sample groups except for basal-like tumours were significantly associated with this pathway. Specifically, CLs exhibited downregulation of many membrane receptors and ECM molecules participating in the activation of the PI3K/Akt/mTOR pathway, while their expression in tumours varied (Tables S6–9). Furthermore, PI3Ks—whose phosphorylation activates AKT—were downregulated in CLs, but not dysregulated in tumours. Heat-shock proteins (HSPs) —also phosphorylating AKT—were upregulated in CLs and normal-like TN tumours; some of them—*HSP90AA1* and *HSP90B1*—were also upregulated in basal-like TN tumours, and one—*HSP90B1*— was upregulated across all sample groups. Finally, *AKT1* was upregulated in CLs, while *AKT3* was downregulated across all sample groups except for basal-like TN tumours, while AKT antagonising factors such as *PTEN* were only downregulated in CLs and normal-like TN tumours. Interestingly, despite differences in pathway activation between tumours and CLs, AKT downstream effectors related to cell cycle, cell survival regulation, and p53 signalling activation were predominantly upregulated across all sample groups but basal-like tumours (Fig. 3). In contrast, members of the mTOR signalling pathway exhibited no dominant dysregulation pattern except *DDIT4*, which was upregulated across all sample groups (Table S6–9).

### Upregulated DEGs in basal-like TN tumours were associated with the antigen processing and presentation pathway

**Figure 4:**
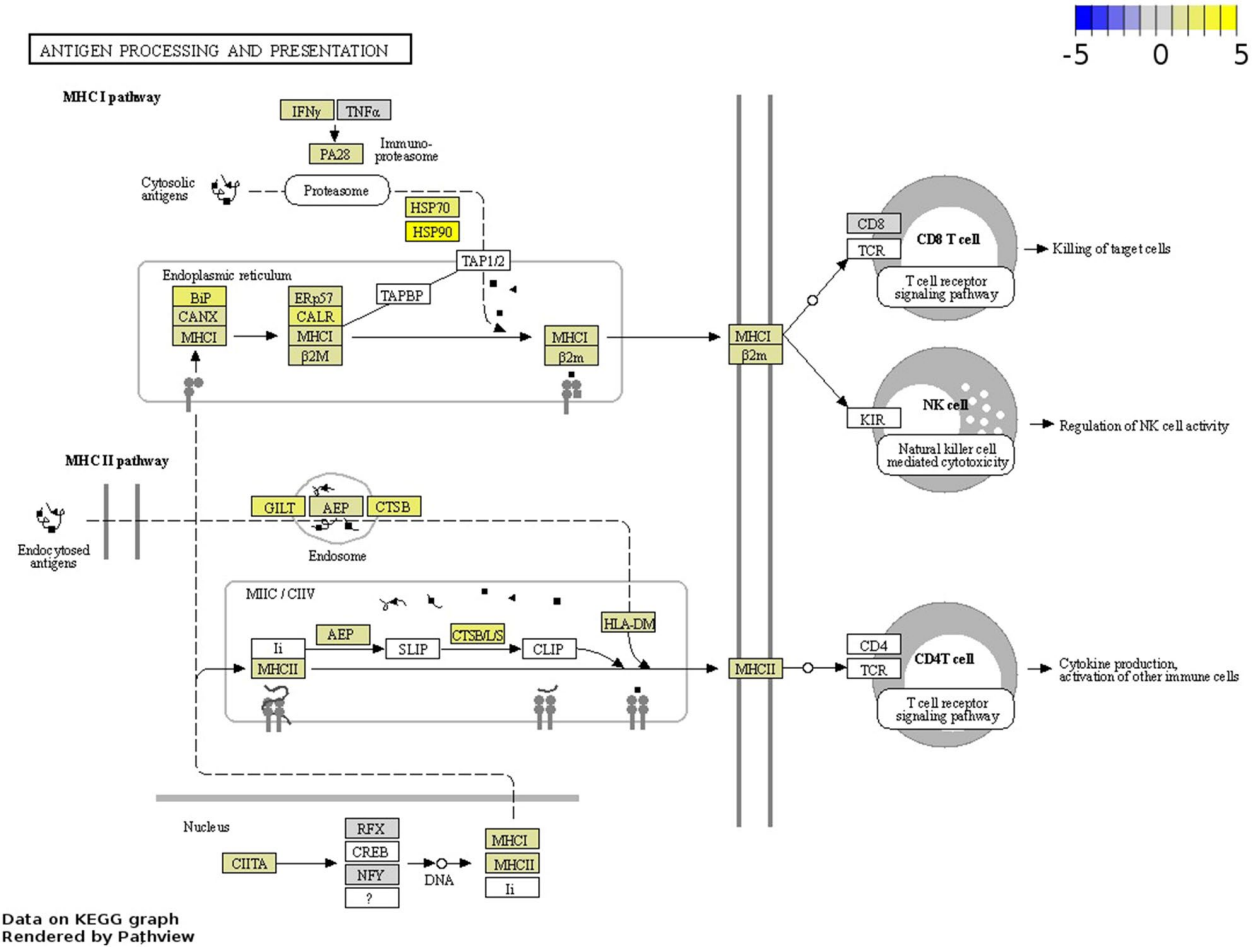
Upregulated genes enriching the Antigen Processing and Presentation pathway (fca04612) in basal-like triple-negative tumours. Gene log_2_ fold change relative to controls is indicated in yellow (upregulated) or blue (downregulated), white boxes represent genes not detected. Data on KEGG graph rendered by “Pathview” (https://pathview.uncc.edu/).

Antigen processing and presentation is pivotal for immune evasion during carcinogenesis and its modulation might be promising for the development of targeted immunotherapies^40^. Upregulated genes basal-like TN tumours were associated with the antigen processing and presentation pathway (fca04612) including genes participating in the MHC I and MHC II pathways—mediating the presentation of endogen cytoplasmic and exogenous (intravesicular) antigens to T and/or NK cells (Fig. 4, and Table S6). Upregulated genes included *CALR* (calreticulin), and several HSPs implicated in tumour immune evasion and critical for maintaining cellular protein homeostasis^41–43^.

### Upregulated GPCRs associated with the neuroactive ligand-receptor interaction pathway are promising oncotarget candidates in normal-like TN tumours

**Figure 5:**
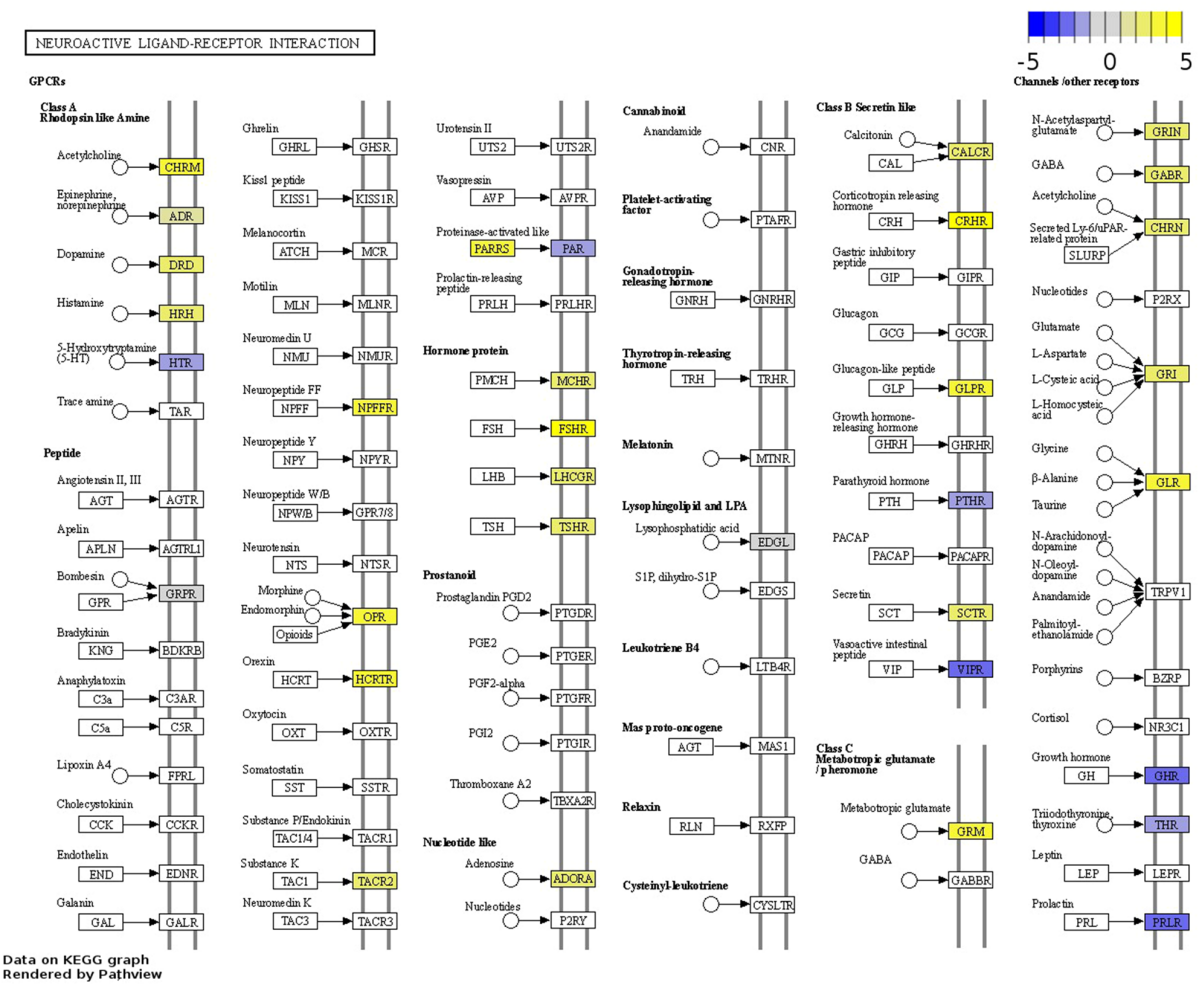
DEGs enriching the Neuroactive Ligand-Receptor Interaction pathway (fca04080) in normal-like triple-negative tumours. Gene log_2_ fold change relative to controls is indicated in yellow (upregulated) or blue (downregulated), white boxes represent genes not detected. Data on KEGG graph rendered by “Pathview” (https://pathview.uncc.edu/).

DEGs in normal-like TN tumours were associated with the neuroactive ligand-receptor interaction pathway. Upregulated DEGs associated with this pathway included several neurotransmitters and neuropeptides receptors (Table S7) triggering cell growth and metastasis through downstream signalling in cancer^44^. Among them, neuropeptide G protein-coupled receptors (GPCRs) —which represent the first gate through which outside signals are transmitted into the cell—have been postulated as possible therapeutic targets^44,45^. Upregulated GPCRs that have been proposed as therapeutic targets in cancer included the adenosine receptors *ADORA1* and *ADORA3*, and important GPCRs for autocrine growth factors in cancer, such as bombesin receptor *NMBR*, and neurotensin receptor *NTSR1*^45,46^. Additional GPCRs reported to promote proliferation and migration of cancer cells included secretin receptor *SCTR*, and lysophosphatidic acid (LPA) receptor *LPAR2*^47,48^. Finally, several upregulated hormone receptors were associated with the neuroactive receptor interaction pathway, including those for melanin-concentrating hormone, thyroid-stimulating hormone, calcitonin, corticotrophin-releasing hormone, follicle-stimulating hormone, luteinizing hormone beta, and glucagon-like peptide (Fig. 5). In contrast to normal-like TN tumours, neurotransmitters receptors were predominantly downregulated in CLs and not dysregulated in other tumour subtypes.

### Components of lysosomes, phagosomes and exosomes might be promising targets in LB fHER2− tumours

**Figure 6:**
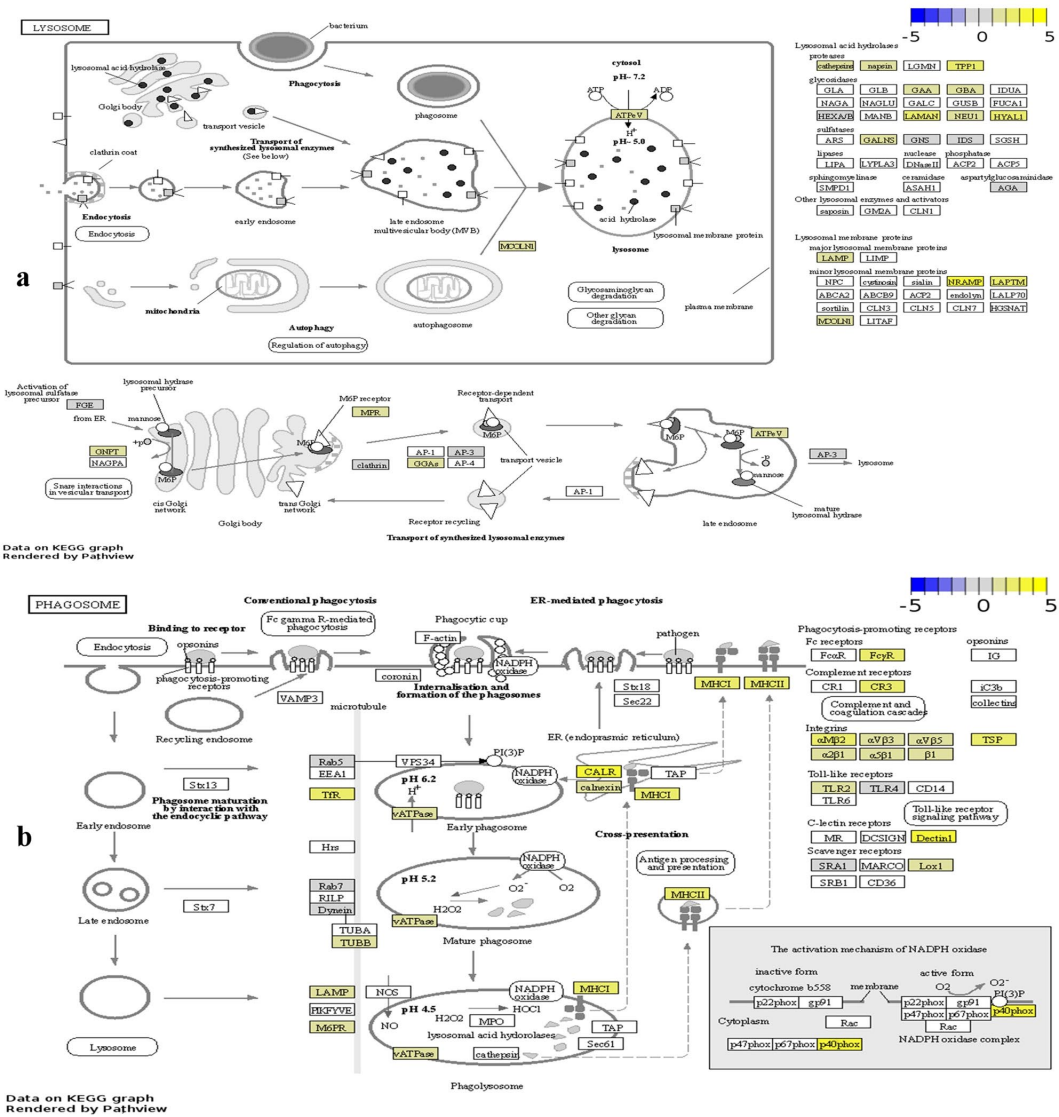
Upregulated genes enriching the (**a**) Lysosome (fca04142), and (**b**) Phagosome (fca04145) pathways in LB *f*HER2- tumours. Gene log_2_ fold change relative to controls is indicated in yellow (upregulated) or blue (downregulated), white boxes represent genes not detected. Data on KEGG graph rendered by “Pathview” (https://pathview.uncc.edu/).

Lysosomes and phagosomes increase energy supply through the recycling of intracellular substances and autophagy and have been reported to enhance cell communication, cancer progression, and metastasis through exosome formation^49–53^. An up-regulation of specific exosome components and the lysosome and phagosome pathways were exclusively detected in LB *f*HER2− tumours (Fig. 6 and Table S8). Accordingly, up-regulated DEGs in this group were annotated with GO terms representing extracellular exosomes, lysosomes, vacuoles, and microtubule cytoskeleton (Fig. S3 and Table S8).

### Upregulated metabolic genes in LB fHER2− tumours and derived CLs were associated with the glycolysis/gluconeogenesis pathway

Notably, genes associated with the glycolysis/gluconeogenesis pathway including LDHA —whose overexpression in cancer is associated with proliferation and malignancy^54–56^— and several glycolytic genes (e.g. *HK1*, *VDAC1*, and *VDAC2*) were only upregulated in LB *f*HER2− tumours and derived CLs (Tables S8 and 9). However, members of this pathway that are possible therapeutic targets such as *SIX1* and some of its targets (e.g., *ENO1*, *PFKL*, and *PGK1*)^57^ were upregulated in all sample types were also upregulated across all sample types. Moreover, several members of the solute carrier family-16—also known as monocarboxylate transporter (MCT) family— catalysing the transport of lactic acid and pyruvate across the plasma membranes were upregulated across different sample groups (Table S6–9).

### Dysregulation of cell lines transcriptome highlights loss of interaction with the tumour microenvironment

We observed differences in the regulation of pathways related to reception and processing of environmental cues between tumours and CLs. For instance, DEGs associated with the ECM-receptor interaction pathway (also involved in PI3K/Akt/mTOR activation) were predominantly upregulated (~ 80%) in LB *f*HER2− tumours, and represented a combination of approximately half up- and downregulated DEGs in TN tumours (Tables S6–8). In contrast, DEGs in this pathway were predominantly downregulated (~ 70%) in CLs. (Table S9). Notoriously, recurrently downregulated DEGs in this pathway represented membrane-, and/or extracellular matrix-associated components shared between the ECM-receptor interaction and the focal adhesion pathways. Additional cell surface components (e.g. laminins and collagens) were also predominantly downregulated in CLs. Similarly, laminins were predominantly downregulated across tumour sample groups among which *LAMA2* and *LAMA3* were downregulated across all sample groups. On the other hand, collagen molecules were up or downregulated across different tumour subtypes and only *COL2A1* was upregulated across all sample groups (Table S10). Additional pathways behind cell adhesion and actin cytoskeleton remodelling were also associated with downregulated DEGs in CLs (e.g. Gap junction, Rap1 signalling, and cell adhesion molecules [CAMs]), as well as, several immune system and endocrine system-related pathways (Table S9). To sum up, cell surface components mediating direct cellular interaction and the transduction of multiple environmental signals including nervous and sensory systems, as well as, endocrine- and immune system-related pathways were significantly associated with downregulated DEGs in CLs highlighting the lack of interaction with structural and non-structural components of the tumour microenvironment (Table S9).

## Discussion

In this study, we comparatively analysed the transcriptome of primary neoplastic samples representing three molecular subtypes of FMCs characterised by poor survival (basal-like TN, DFI: 3.35 ± 1.71 months, CSS: 3.73 ± 1.67 months], normal-like TN [DFI: 7.22 ± 4.87 months, and CSS: 9.34 ± 4.28 months], and LB *f*HER2− [DFI: 12 ± 5.9, and CSS: 14.3 ± 4.2 months]) aiming to identify common and exclusive therapeutic targets, prognostic markers and clinically relevant gene expression changes eliciting cancer progression. Furthermore, we analysed two LB *f*HER2− derived CLs to uncover similarities and differences that might impact their ability to model distinct aspects of the tumour dysregulation. Limitations of this study include the small sample size, the lack of representation of molecular subtypes with better prognosis, and the poor quality of the RNA isolated from FFPE samples, reflected by the higher similarity observed between FT and CL samples. Furthermore, only one FT sample was included in the control group. Additionally, tumour tissues include a certain amount of normal cells, and usually display clonal heterogeneity, which in combination with patient-specific effects might impact the number of detectable dysregulated genes. Therefore, as RNA was indistinctively isolated from all cellular subpopulations included in the tumour sample, a contamination with healthy mammary tissue is possible.

Top up- and downregulated DEGs were similarly dysregulated across all sample groups. However, only *RACGAP1* was among the top upregulated DEGs across all sample groups, and *NEK2* across TN subtypes. *RACGAP1* and *NEK2* have a pivotal role during cytokinesis and are known prognostic markers in HBC: high expression levels of these genes are correlated with high proliferation and poor prognosis^58–61^. Importantly, metabolic-related genes including proposed therapeutic targets in different human cancers^42,62–66^ were among the top upregulated DEGs (e.g. *FADS2, HSPH1, VLDLR*, *TF,* and *ASNS*) across different sample groups, emphasizing the importance of new therapeutic strategies targeting metabolic reprogramming. In line with the picture emerging from the top upregulated DEGs, recurrently upregulated genes across all sample groups were also associated with cell cycle- and metabolic-related pathways. Among them, we identified prognostic and therapeutic targets (e.g. *CCNB1*, *CCNB2*, *CDK1*, *CDK4*, *GTSE1*, *MCM4*, and *MCM5*) implicated in DNA replication, cell cycle, and signalling pathways controlling proliferation (e.g. p53, and PI3K-AKT).

The main recurrent dysregulated pathways in this study were the p53 and PI3K-AKT, whose interactions play a significant role in the determination of cell death/survival and have been previously reported as important in FMCs^67–72^. The observed concurrent up-regulation of *TP53* and downstream effectors *CCNB1*, *CCNB2*, *CDK1*, and *CDK4*—normally inhibited by genes whose expression is promoted by p53—suggests as previously documented on FMCs the presence of *TP53* mutations or post-transcriptional modifications resulting in the loss of p53 tumour suppressor activity^70–72^. Furthermore, in tumours retaining wild-type *TP53*, p53 function might still be impaired, with *MDM2* and *MDM4* being the most important regulators of its activity^29^. However, in this study *MDM2* and *MDM4* were only dysregulated in CL samples. Accordingly, future research should focus on characterising mutations, post-transcriptional modifications, and possibly targeting the mutant p53 protein and/or restoring wild-type p53 activity^30^. The more extensive dysregulation of p53 signalling pathway observed in CLs might facilitate cell survival, replication, and adaptation to culture conditions, as gene dysregulation behind p53 signalling presumably mediates different responses aiming to protect neoplastic cells against different transformation-stress stimuli^28^. As in the p53 pathway, AKT downstream effectors related to proliferation and survival remained upregulated across all sample groups. Moreover, in contrast to previous reports in HBC^73–75^, *PIK3CA* and other PI3Ks activating AKT as well as *AKT1* and *AKT3* were downregulated or not differentially expressed in tumours, suggesting that subtypes evaluated here are not likely to respond to therapies targeting PI3Ks and AKT inhibitors.

Upregulated DEGs included several proposed prognostic markers and therapeutic targets in cancer-relevant for DNA replication, cell cycle- and gene expression regulation. For instance, *MCM4* and *MCM5* are essential for the initiation of genome replication^76,77^, and were recurrently upregulated across all sample groups. Additional members of the MCM complex (i.e. *MCM2*, *MCM3*, *MCM6*, and *MCM7*) were upregulated in all sample groups but basal-like TN tumours. These results are in line with previous studies on HBC in which independent or simultaneous upregulation/overexpression of MCM members was correlated with poor outcomes^78–80^, suggesting a role of MCM members as biomarkers of poor prognosis and possible therapeutic targets in FMCs. Other genes involved in regulating the cell cycle that were recurrently upregulated across all sample groups included *GTSE1*, whose upregulation has been associated with poor prognosis in several human malignancies^33–35^, and is believed to enhance metastasis in human TNBC^81^ and to regulate cancer progression by affecting p53 function^32,34^. *GTSE1* knockdown reduces cell proliferation and increases sensitivity to radio- and chemotherapy in in vitro models by blocking the expression of *LHDA*^82–85^. Hence, it is a potential therapeutic target in FMCs. Furthermore, we identified upregulated DEGs in TN tumours including drug targets histone deacetylase enzymes (e.g. *HDAC1, HDAC2, and HDAC11*) implicated in the epigenetic regulation of gene expression^86,87^. Approved HDAC inhibitors (HDACis) for human cancer include vorinostat, romidepsin, panobinostat, and belinostat-like. Furthermore, several HDACis have been tested in vitro in FMCs^88^, and in vitro and in vivo different canine tumours, documenting antineoplastic activity, good tolerability, and clinical response in some cases^89,90^.

Considering the identification of recurrent DEGs across all sample groups associated with several metabolic pathways, FMCs might be susceptible to therapies targeting cancer metabolic rewiring^91,92^. Among possible metabolic targets, *HSP90B1* was upregulated across all sample groups. HSP90B1 is reported to enhance apoptosis evasion and its overexpression is correlated with metastasis and poor prognosis in HBC^93–95^. Furthermore, we detected an upregulation in CLs and across different tumour subtypes of heat-shock proteins (e.g. *HSP90AA1*, *HSP90AB1*, *HSP90B1*, *HSPA5*, *HSPA8*, *HSPD1*, and *HSPH1*) associated with malignancy and implicated in the stabilization of important cancer-related proteins (e.g. KIT, MET, BRAF and AKT), the formation of exosomes, and the presentation of tumour antigens^43,96–98^. The upregulation of HSP90 family members is targetable with small molecule inhibitors (e.g., ganetespib, 17-AAG, and STA-1474). Moreover, STA-1474 has been tested in dogs with spontaneous tumours demonstrating tolerability, and biological activity against several neoplasms^99,100^. Besides HSP90 family members, molecules targeting *HSPA5* (upregulated across all sample groups) master regulator of endoplasmic reticulum homeostasis have also been reported as a therapeutic strategy in human melanoma^101,102^. Finally, several monocarboxylate transporters (MCTs) implicated in the export of lactic acid and pyruvate across the plasma membrane were upregulated in different sample groups. Among them, the Solute Carrier Family 16 Member 3 *SLC16A3* (MCT4) was upregulated across all sample groups. MCT4 supports tumour proliferation, and has been associated with poor prognosis and proposed as therapeutic target in different human cancers^103–105^.

Cell–cell and cell-matrix interactions eliciting cell proliferation and migration are determined by the tumour microenvironment characteristics, which are to great extent affected by dysregulation of ECM components^106,107^. In this study, ECM molecules in tumours included both up- and downregulated genes—some of them being exclusively dysregulated in specific molecular subtypes. Dysregulation of specific ECM components might enhance local invasion and metastasis. For instance, the collagen network reorganization might facilitate cell migration, which has been reported as a predictive factor of worst outcome in FMCs^108^. Among all collagen molecules, only collagen type II alpha 1 chain (*COL2A1*) was found to be upregulated across all sample groups. This in agreement with a previous report documenting a positive association between COL2A1 protein levels and canine mammary tumours development^109^. On the other hand, *COL2A1* overexpression is associated with poor clinical response and cell culture resistance to combinatorial HER2 and PI3K inhibition in HBC^110^. Furthermore, *COL2A1* mutations are associated with chondrosarcoma development in humans^111–113^, and it was recently reported as a biomarker of melanoma repopulating cells exerting cancer stem-like cell properties^114^. Considering the emerging roles of *COL2A1* in cancer dysregulation, findings of this study support the evaluation of *COL2A1* role on FMCs progression, as well as, possible prognostic and therapeutic implications.

To identify DEGs that might be relevant to the development of targeted panels or to the design of subtype-specific targeted therapies, we analysed the function of the DEGs in each tumour subtype. The studied subtypes displayed distinctive groups of DEGs that might contribute to molecular events characterising carcinogenesis. For instance, only LB *f*HER2− tumours exhibited upregulated genes in the lysosome and phagosome pathways. Hence, it is plausible that this subtype might be especially susceptible to blocking the formation of autophagolysosomes, as well as, other autophagy-targeted therapies^53^. Furthermore, LB *f*HER2− sample groups (tumours and CLs) displayed specific upregulation of several glycolytic genes including *HK1*, *VDAC1*, and *VDAC2*. Similarly, *LDHA*—which converts pyruvate to lactic acid—was only upregulated in LB *f*HER2− sample groups, highlighting a particular susceptibility to glycolytic inhibitors, and the potential of the CLs described here for testing this therapeutic strategy. On the other hand, normal-like TN tumours exhibited upregulated genes in the Neuroactive ligand-receptor interaction pathway, including major components of the superfamily of rhodopsin-like G protein-coupled receptors (GPCRs). GPCRs function as autocrine growth factors in cancer and have been suggested as suitable therapeutic targets^45,47,48^. Finally, basal-like TN tumours showed an exclusive upregulation of several HCM class I related heat-shock proteins (HSPs) and genes associated with the Antigen Processing and Presentation pathway, suggesting a more important role of immune surveillance evasion and specific sensibility to inhibitors of the proteasome, HSPAs, and cancer immunotherapy^40,42,115^.

To sum up, our results suggest that upregulation of genes related to cell cycle control and metabolic reprogramming remain similar after culture establishment and subculturing. In agreement with previous studies^116–118^, genes that would normally mediate cellular adhesion and prevent migration were predominantly downregulated in both tumours and CLs. However, downregulated genes in CLs included signalling molecules and membrane receptors such as protein kinases and phosphatases, their substrates, and various adapter proteins. In particular, the extensive downregulation of cell surface components and signalling pathways mediating cell–cell and cell–matrix cross-talks observed in CLs highlights cells disconnection with the cellular and non-cellular components of the tumour microenvironment and points out limitations of immortalised cells to model complex interactions between the tumour and the hosting organism during cancer development and progression. As previously described^117,119,120^, CLs evaluated in this study might fail to address the role of the immune, endocrine, and neurologic systems stimuli during cancer progression. The higher number of DEGs—particularly, downregulated genes— detected in CLs in comparison to tumours most likely evidences adaptation to culturing conditions, but may also partially reflect clonal selection and poor tissue sample quality.

The purpose of this research was to unravel biologically relevant gene expression differences and similarities across three molecular subtypes (basal-like TN, normal-like TN, and LB *f*HER2−) of feline mammary tumours, and among primary lesions and established cell lines. Pathways and DEGs characterising cell cycle control and metabolic reprogramming are recurrent across different subtypes and remain similar in derived cellular models. However, a higher number of downregulated genes specifically enriching cell components and pathways involved in cellular communication characterised the dysregulation of CLs and might impact in vitro modelling. Importantly, we identified recurrent and subtype-specific dysregulated genes susceptible to different targeted therapies including histone deacetylases, heat-shock proteins, metabolic-related genes, and genes controlling centrosome disjunction and cell cycle. Moreover, molecular subtype-specific DEG sets associated with lysosomes, phagosomes and exosomes formation in LB *f*HER2− tumours; GPCRs in normal-like TN tumours; and antigen processing and presentation in basal-like TN tumours highlight the importance of interaction with the tumour microenvironment during FMCs progression and provide an avenue for the development of molecular subtype targeted panels and possible subtype-tailored therapeutic approaches.

## Methods

### Animals and samples

Primary neoplastic samples (CL, FF, and FT) were collected from female cats diagnosed with FMCs, surgically treated and followed-up for at least two years at the Small Animal Clinic, University of Veterinary Medicine Hannover. Patients included in this study are part of a larger cohort in which mass-parallel genomic sequencing was used to characterise copy-number variations^10^. Moreover, copy-number variations affecting one of the CLs included in this study (TiHo-0906) were also characterised using mass-parallel genomic sequencing in a previous publication from our group^26^. Patients included in this study were followed up during a two-year postoperative period, animals without recurrence or alive at the end of the study were excluded from DFI and cancer-specific survival analysis, respectively. All samples included in this study were obtained from tissue specimens surgically retrieved during medically necessary treatment and diagnosis after owner’s written approval. Therefore, in accordance with the German Animal Welfare Act this study was not considered an animal experiment and ethical approval was not required.

### Cell lines culture and RNA extraction

TiHo-1403 and TiHo-0906 were isolated from the primary tumours of two animals included in this study (Fig. S1) and were established and cultured as previously described^26^.Cultures with growth medium (medium 199 with 10% foetal bovine serum [FBS, Biochrom], and 2% penicillin–streptomycin) were incubated in a humidified atmosphere (5% CO_2_) at 37 °C. To obtain pellets for RNA isolation, cells were detached using a dissociating agent (TrypLETM, Gibco). After centrifugation (1000 g for 10 min) of cell suspensions, the supernatant was discarded to obtain cell pellets. Cell pellets were resuspended in 1 mL freezing medium (medium 199 with 20% FBS, 2% penicillin–streptomycin and 10% DMSO [AppliChem]) and transferred into vials for cryogenic storage. Vials were preserved in a − 80 °C freezer during 24 h, and afterward, stored in a − 150 °C freezer until RNA extraction. For nucleic acids extraction, samples containing 4 × 10^6^ cells (TiHo-0906 p7 and p77, and TiHo-1403 p10) were homogenized using QIAshredderTM columns (QIAGEN). For all samples types (FFPE, FT and CL), RNA extraction was performed using the AllPrep DNA/RNA Mini Kit (QIAGEN) according to manufacturer’s instructions. RNA yields and purity (260/280 ratio) were measured with the Synergy 2 plate-reader (BioTek).

### RNA-seq

Sequencing libraries were prepared using the NEBNext Ultra Library Prep Kit for Illumina. Sequencing was conducted on an Illumina NextSeq500, single-reads with 75 bp were generated. Reads were mapped to the feline genome (FelCat5) using BWA^121^.

Counts from sequencing libraries corresponding to different passages of the TiHo-0906 cell line were added together.

### Differential expression analysis

Differential expression analysis was conducted with the DESeq2 R package (version 1.24)^122^. Only protein-coding genes were considered for the analysis. Samples were divided into five sample groups: normal-like TN primary neoplastic samples, LB *f*HER2− primary neoplastic samples, basal-like TN primary neoplastic samples, LB *f*HER2 negative-derived cell lines, and healthy mammary tissue samples. Expression was compared between each of the primary neoplastic or cell line sample groups and the healthy mammary tissue sample group. Genes that had an adjusted P-value (false discovery rate, FDR) smaller than or equal to 0.05 and exhibited a fold-change greater than or equal to 2 (or less than or equal to 0.5) were considered differentially expressed.

The “top differentially expressed genes” (“top DEGs”) were defined for each sample group as the set consisting of the 5 upregulated and 5 downregulated DEGs with the smallest FDR.

### Expression values

Expression values were obtained by applying the regularized logarithm transformation implemented in the rlog() function of the DESeq2 R package^122^ to the raw read counts; no experimental design (“design =  ~ 1”) was used for this purpose.

### Functional and pathway analysis

Functional analysis of DEGs was performed with DAVID (Database for annotation, visualization and integrated discovery tool)^123,124^. Specifically, the following ontologies and databases were considered: GOTERM_MF_ALL, GOTERM_BP_ALL, GOTERM_CC_ALL, GOTERM_MF_DIRECT, GOTERM_MF_FAT, GOTERM_CC_FAT, and KEGG pathways. The DAVID default list of corresponding genome-wide genes for the species was used as background. Terms with a false discovery rate (FDR) equal to or less than 5% were considered significant.

PATHVIEW was used to visualise enriched KEGG pathways (https://pathview.uncc.edu)^125,126^. Furthermore, all analyses were performed for total DEGs (up- and downregulated genes together), and up-, and downregulated genes separately. REVIGO was used to summarize and visualise the enriched GO terms^127^.

### Statistics

Descriptive statistics, and survival analysis were performed using the statistical software SPSS (IBM SPSS Statistics for Windows, Version 23.0. Armonk, NY, USA). DFI and CSS were calculated as months from surgery to tumour recurrence (local or distant), and to death, respectively. Cat deaths without recurrence were censored from DFI analysis, cats alive at the end of the study period (24 months) and animals that died due to non-tumour related causes were censored from cancer-specific OS analysis. All data is displayed as mean ± standard deviation unless indicated otherwise. For all statistical analyses, a *p* ≤ 0.05 was considered significant.

## Supplementary Information

Supplementary Information.

## Data Availability

The datasets generated during and/or analysed during the current study are available from the corresponding author on reasonable request.
