# Supplementary material for: Transcription profiling of feline mammary carcinomas and derived cell lines reveals biomarkers and drug targets associated with metabolic and cell cycle pathways
Source: Sci Rep. 2022 Oct 11;12:17025. doi: 10.1038/s41598-022-20874-5 (PMC9553959; doi:10.1038/s41598-022-20874-5)
Supplement: Supplementary file 1 — Supplementary Information. [file 41598_2022_20874_MOESM1_ESM.pdf]

# Transcription profiling of feline mammary carcinomas and derived cell lines reveals biomarkers and drug targets associated with metabolic and cell cycle pathways

José Luis Granados-Soler, Leila Taher, Julia Beck, Kirsten Bornemann-Kolatzki, Bertram Brenig, Verena Nerschbach, Fernando Ferreira, Johannes Junginger, Marion Hewicker-Trautwein, Hugo Murua Escobar, Ingo Nolte

## Supplementary Material

### *Supplementary Figures*

**Supplementary figure 1.** Euclidean distances between pairs of samples.
**Supplementary figure 2.** Semantic analysis of GO terms associated with recurrent up-regulated genes across all sample types.
**Supplementary figure 3.** Semantic analysis of cellular component GO terms associated with recurrent up-regulated genes in LB fHER2- tumours.

### *Supplementary Tables*

**Supplementary table 1.** Characteristics of cases included in this study.
**Supplementary table 2.** Patients' follow-up.
**Supplementary table 3.** Recurrent genes differentially expressed across all sample groups.
**Supplementary table 4.** Top DEGs across all sample groups.
**Supplementary table 5.** Recurrent DEGs across all sample types associated with selected KEGG pathways.
**Supplementary table 6.** Differentially expressed genes in basal-like triple negative tumours associated with selected KEGG pathways.
**Supplementary table 7.** Differentially expressed genes in normal-like triple negative tumours associated with selected KEGG pathways.
**Supplementary table 8.** Differentially expressed genes in Luminal B *f*HER2 negative tumours associated with selected KEGG pathways and GO terms.
**Supplementary table 9.** Differentially expressed genes in Luminal B *f*HER2 negative-derived cell lines associated with selected KEGG pathways.
**Supplementary table 10.** Collagen molecules expression across sample groups.

# Supplementary Figures

**Supplementary figure 1. Euclidean distances between pairs of samples.** Cell line and frozen tissue samples clustered together (Group 1 blue), while all FFPE samples formed a separate cluster (Group 1 green), both clusters included a mixture of control and neoplastic samples. FT, frozen tissue; FFPE, formalin-fixed paraffin-embedded; CL, cell line; and TN, triple-negative; paired CL/OT, paired cell line/original tumour; paired T/C, paired Tumour/Control. *Normal like TN, ** basal-like TN, and †, and #, original tumour/derived cell line. Paired samples neoplastic/control samples identified as 1 to 7.

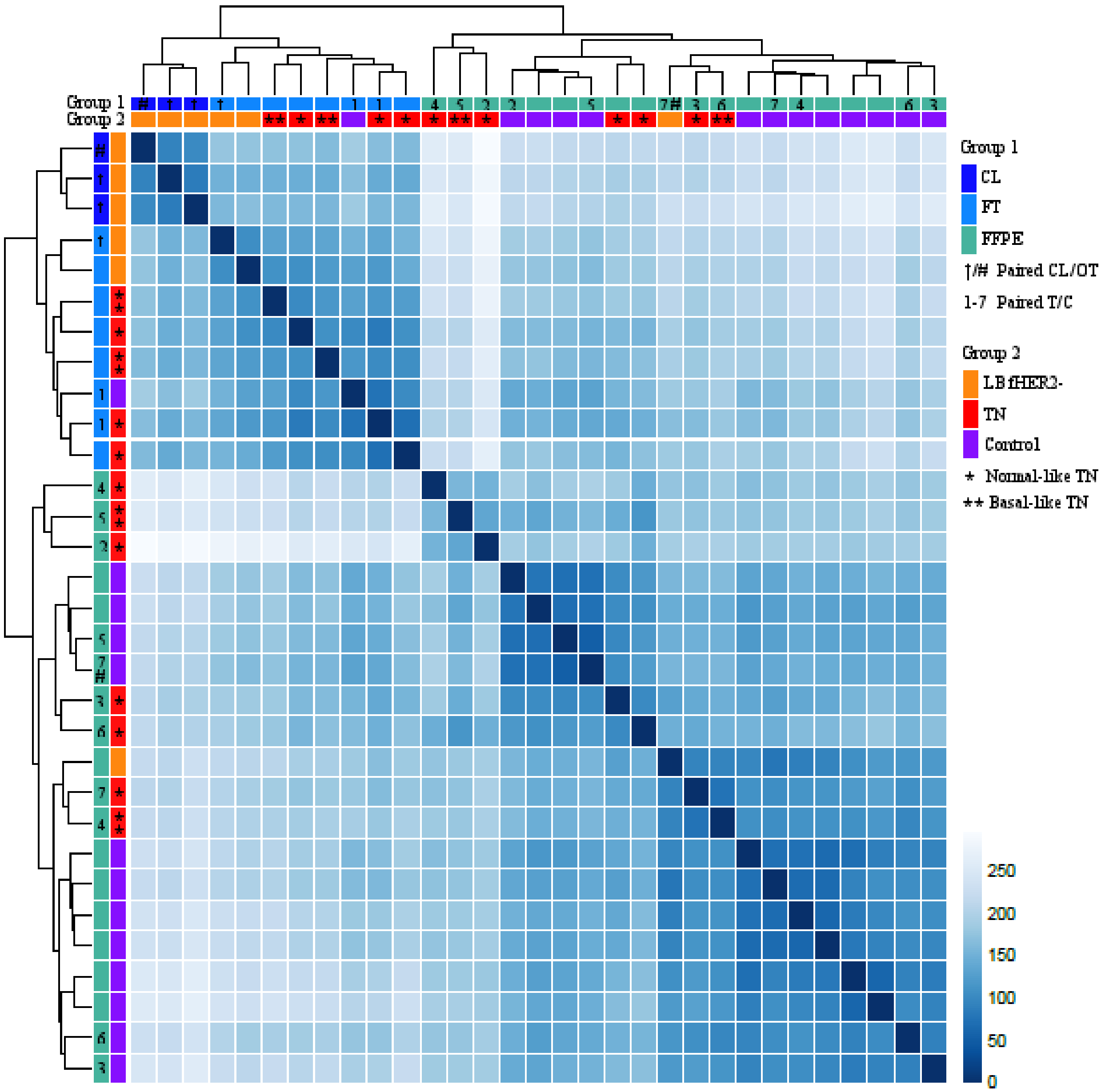

**Supplementary figure 2. Semantic analysis of GO terms associated with recurrent up-regulated genes across all sample types**. GO terms classified as a) biological process, and b), and molecular function. Semantically similar GO terms remain close together in the plot but the semantic space units have no intrinsic meaning. Circle colour indicates the negative log10 FDR value; circle size indicates the frequency of the GO terms in the underlying GO database. Data analysed with REVIGO (http://revigo.irb.hr/).

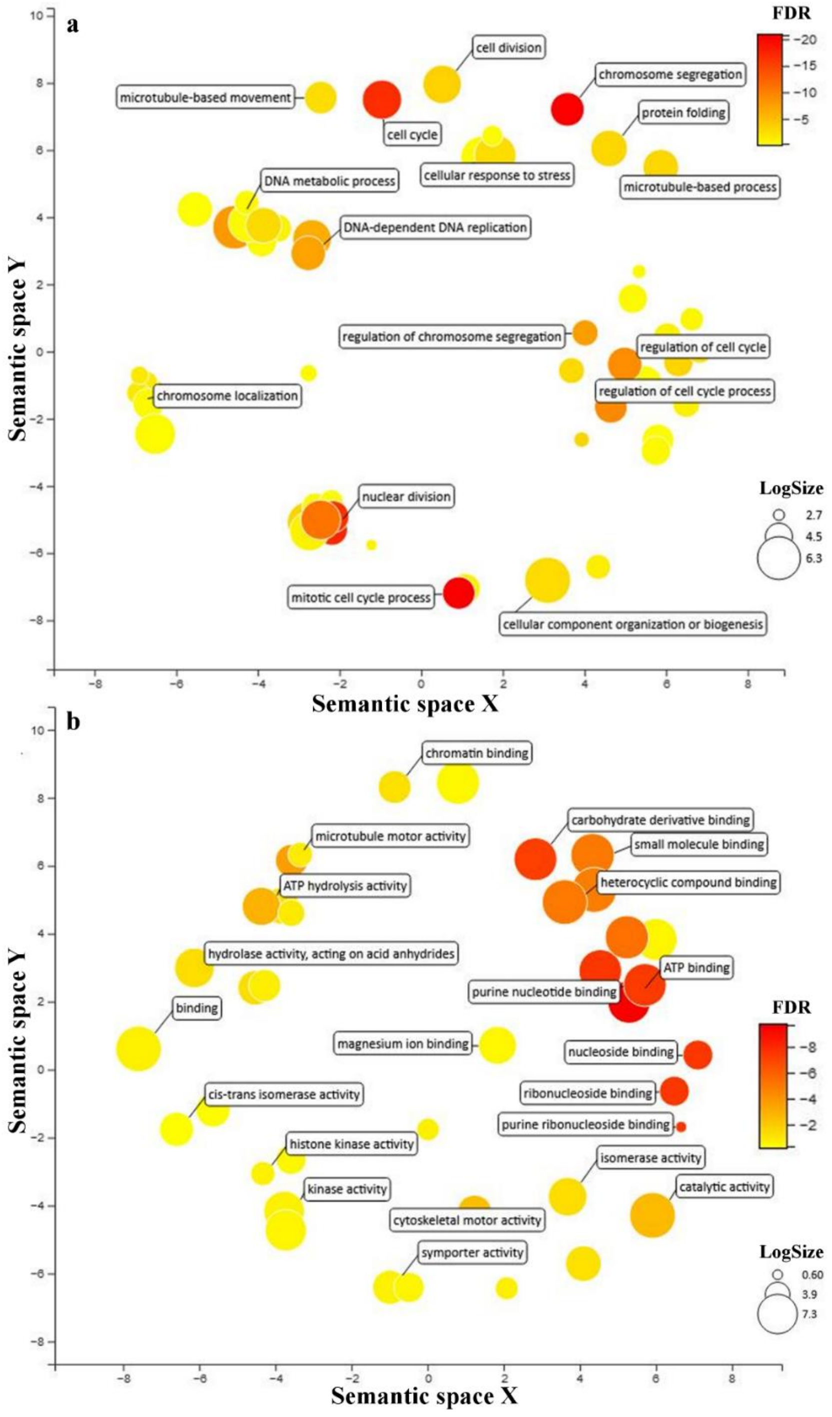

**Supplementary figure 3. Semantic analysis of cellular component GO terms associated with recurrent up-regulated genes in LB *f*HER2- tumours**. Semantically similar GO terms remain close together in the plot but the semantic space units have no intrinsic meaning. Circle colour indicates the negative log10 FDR value; circle size indicates the frequency of the GO terms in the underlying GO database. Data analysed with REVIGO (http://revigo.irb.hr/).

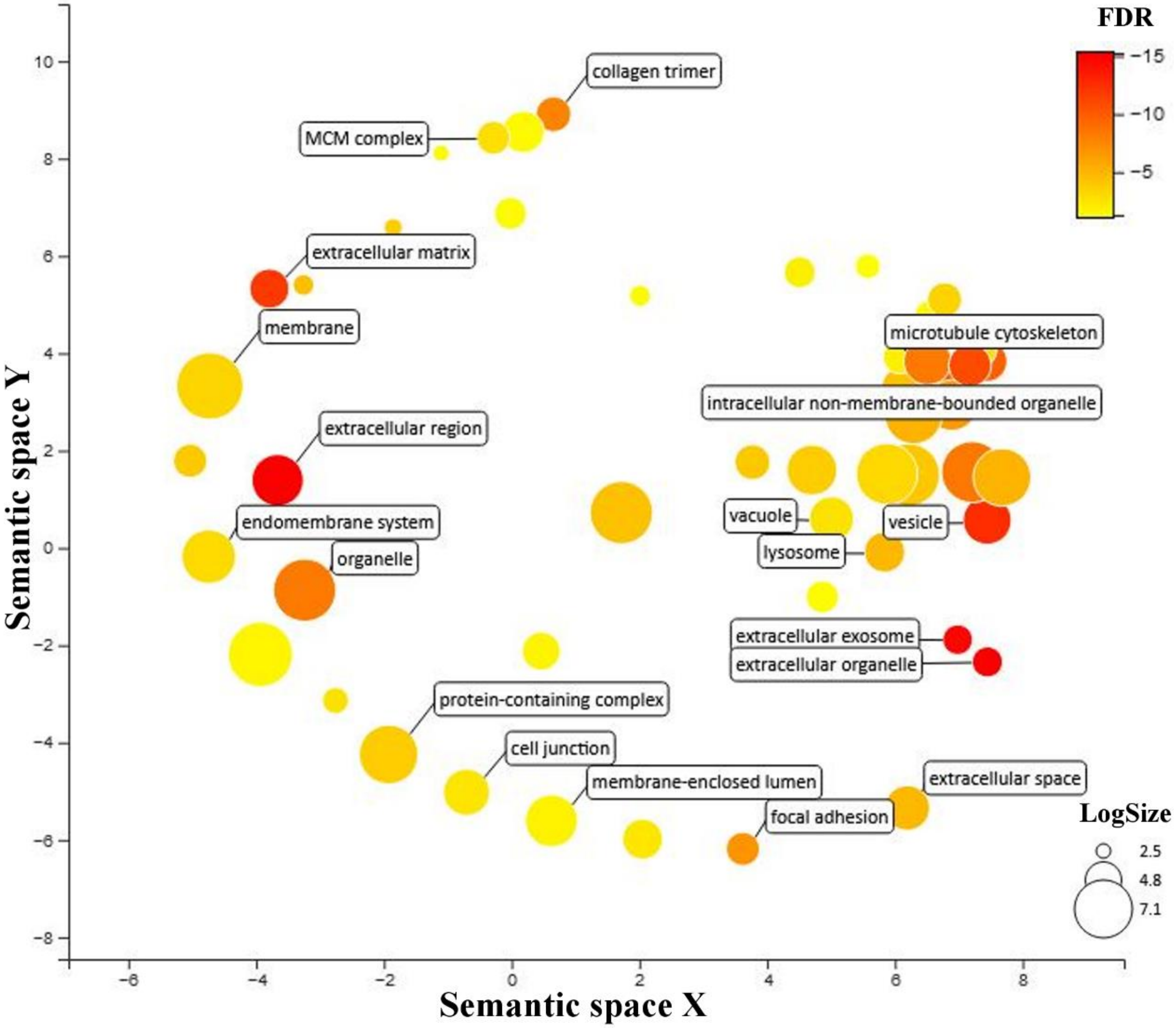

## Supplementary tables

**Supplementary table 1.** Characteristics of cases included in this study.

| Feature | Percentage of cases (n=15) |
|---|---|
| **Patients features** | |
| ***Age*** | |
| ≤ 10 years | 12 (80%) |
| ≥ 10 years | 3 (20%) |
| ***Breed*** | |
| Domestic shorthair | 8 (53.33%) |
| Siamese | 1 (6.67%) |
| Norwegian Forest cat | 2 (13.33%) |
| Chartreux | 2 (13.33%) |
| Abyssinian | 1 (6.67 %) |
| British Shorthair | 1 (6.67 %) |
| ***Neutered*** | |
| No | 10 (66.67%) |
| Yes | 5 (33.33%) |
| ***Affected mammary complex*** | |
| Thoracic | 2 (13.33%) |
| Caudal abdominal | 6 (13.33%) |
| Inguinal | 7 (15.56%) |
| ***Tumour size*** *[1]* | |
| ≤2 cm | 2 (13.33%) |
| 2-3 cm | 6 (40%) |
| ≥3 cm | 7 (46.76%) |
| ***Clinical stage*** *[1]* | |
| I | 1 (6.67%) |
| II | 2 (13.33%) |
| III | 12 (80%) |
| **Tumours features** | |
| ***Lymph-node invasion*** | |
| Negative | 4 (26.67%) |
| Positive | 11 (73.33%) |
| ***Lymphovascular invasion/neoplastic emboli*** | |
| Negative | 8 (53.33%) |
| Positive | 7 (46.67%) |
| ***EE grading system*** | |
| II | 6 (40%) |
| III | 9 (60%) |
| ***MMEE grading system*** | |
| I | 4 (26.67%) |
| II | 9 (60%) |
| III | 2 (13:33%) |
| ***Mills-2015 grading system*** | |
| II | 6 (40%) |
| III | 9 (60%) |
| **ER *status*** [2-4] | |
| Negative | 13 (86.67%) |
| Positive | 2 (13.63%) |
| **PR *status*** [2-4] | |
| Negative | 11 (73.33%) |

| | |
|---|---|
| Positive | 4 (26.67%) |
| ***f*HER2 *score*** [4-7] | |
| 0 (negative) | 2 (13.33%) |
| 1+ (negative) | 10 (66.67%) |
| 2+ (equivocal) | 3 (20%) |
| ***fHER2 CNGs (Log2 ≥ 0.5)*** [8] | |
| Absent | 12 (80%) |
| Present | 3 (20%) |
| ***f*HER2 *status after HER2-associated CNGs detection*** [8] | |
| Negative | 15 (100%) |
| **CK5/6 *status*** [9] | |
| Negative | 16 (84%) |
| Positive | 3 (16%) |
| **Ki-67 *index*** [10] | |
| Low | 5 (26%) |
| High | 14 (74%) |
| ***St. Gallen molecular subtype*** | |
| LB *f*HER2- | 3 (20%) |
| Normal-like TN | 8 (53.33%) |
| Basal-like TN | 4 (26.67%) |
| EE, Elston and Ellis [11, 12]; MMEE, mitotic-modified EE [13, 14]; Mills-2015, novel histological malignancy grading system for evaluation of FMCs [13, 14]. I: well differentiated, II: moderately differentiated, and III: poorly differentiated. Ki-67 index, low ≤14%, and high ≥14% [10]. LB *f*HER2-, luminal B *f*HER2 negative; and TN, triple-negative. *f*HER2 CNGs: FCA E1 38–44 Mb. Information presented for selected cases was adapted from Granados-Soler *et al*. (2020) [8]. | |

**Supplementary table 2.** Patients' follow-up.

| Variable | All cases together (n = 15) | LB *f*HER2- (n = 3) | Normal-like TN (n = 8) | Basal-like TN (n = 4) |
|---|---|---|---|---|
| ***Local recurrence*** | | | | |
| No | 6 (40%) | 1 (33.33%) | 2 (25%) | 1 (25%) |
| Yes | 9 (60%) | 2 (66.67%) | 6 (75%) | 3 (75%) |
| ***Distant metastases*** | | | | |
| No | 6 (32%) | 1 (33.33%) | 1 (12.5%) | 0 |
| Yes | 13 (68%) | 2 (66.67%) | 7 (87.5%) | 4 (100%) |
| ***Tumour-related death*** | | | | |
| No | 6 (32%) | 1 (33.33%) | 1 (12.5%) | 0 |
| Yes | 13 (68%) | 2 (66.67%) | 7 (87.5%) | 4 (100%) |
| ***Alive at the end of the study period*** | | | | |
| No | 15 (79%) | 1 (33.33%) | 0 | 0 |
| Yes | 4 (21%) | 2 (66.67%) | 8 (100%) | 4 (100%) |
| Survival information from selected cases was adapted according to sample size from Granados-Soler *et al*. (2020) [8]. | | | | |

**Supplementary table 3.** Recurrent genes differentially expressed across all sample groups.

| ***Upregulated DEGs*** |
|---|
| *ACOT7*, *ADAMTS7*, *ADCK2*, *AKR1A1*, *ANLN*, *ARHGAP11A*, *ARHGDIA*, *ASF1B*, *ATP13A2*, *AURKA*, *BOP1*, *BUB1*, *BUB1B*, *CALR*, *CCDC106*, *CCNB1*, *CCNB2*, *CCNB3*, *CCNF*, *CCT5*, *CCT7*, *CDC25B*, *CDC6*, *CDCA3*, *CDCA8*, *CDK1*, *CDK16*, *CDK2AP1*, *CDK4*, *CDT1*, *CENPA*, *CENPE*, *CENPL*, *CENPM*, *CENPT*, *CEP55*, *CFAP47*, *CHAF1A*, *CHEK1*, *CHTF18*, *CKAP2*, *CKAP2L*, *CLSPN*, *CLSTN3*, *COL2A1*, *COL9A3*, *DCHS2*, *DDIAS*, *DDIT4*, *DEGS1*, *DEPDC1*, *DLGAP5*, *DNASE1L1*, *DOK1*, *DSCC1*, *DSN1*, *DYNC2I2*, *E2F1*, *E2F2*, |

| |
|---|
| *E2F7*, *ECE2*, *EFNB1*, *EMG1*, *ENO1*, *ENSFCAG00000000841*, *ENSFCAG00000009370*, *ENSFCAG00000021897*, *ENSFCAG00000029533*, *ENSFCAG00000030093*, *ESPL1*, *EXO1*, *FADS1*, *FADS2*, *FAM186A*, *FEN1*, *FIBIN*, *FKBP10*, *FKBP4*, *FN1*, *FOXM1*, *GALE*, *GLIS2*, *GLRX5*, *GPR19*, *GTSE1*, *HDGF*, *HMBS*, *HMMR*, *HSP90B1*, *HSPA5*, *HSPA8*, *HSPD1*, *HSPH1*, *HYOU1*, *INCENP*, *INTS1*, *IQGAP3*, *ISG20*, *KAT2A*, *KDELR3*, *KIF14*, *KIF15*, *KIF18B*, *KIF22*, *KIF2C*, *KIF4A*, *KIFC1*, *KNL1*, *KPNA2*, *LDLR*, *LMNB1*, *LMNB2*, *LOXL2*, *LRRC59*, *LTBR*, *MARS1*, *MCM4*, *MCM5*, *MFSD12*, *MIIP*, *MIS18A*, *MLEC*, *MMP3*, *MOGS*, *MRPL10*, *MTHFD2*, *MYBL2*, *NACC1*, *NCAPH*, *NCKIPSD*, *NCR3LG1*, *NCS1*, *NDC80*, *NEK2*, *NES*, *NPM3*, *NUF2*, *ORC6*, *PARP1*, *PCK2*, *PCNA*, *PDIA4*, *PDIA6*, *PFKL*, *PFKP*, *PGK1*, *PHLDA1*, *PIF1*, *PIMREG*, *PKM*, *PKMYT1*, *PLOD1*, *POLD1*, *PRKCSH*, *PRR11*, *PTTG1*, *RACGAP1*, *RCC2*, *RHNO1*, *RPS6KA4*, *RRM2*, *SET*, *SGSH*, *SHMT2*, *SIX1*, *SKA1*, *SLC16A3*, *SLC17A9*, *SLC1A4*, *SLC1A5*, *SLC25A39*, *SLC25A5*, *SLC2A1*, *SLC2A6*, *SLC35A2*, *SLC6A8*, *SPAG5*, *SPC25*, *SPDL1*, *SREBF2*, *ST3GAL4*, *STIP1*, *TACC3*, *TCF19*, *TIMELESS*, *TK1*, *TMED3*, *TMEM104*, *TONSL*, *TOP2A*, *TOR1B*, *TPX2*, *TRAIP*, *TRIP13*, *TROAP*, *TTC9C*, *TTK*, *UBE2C*, *UBE2S*, *UBE2T*, *UHRF1*, *WNT7B*, *ZWINT* |
| ***Downregulated DEGs*** |
| *ABCA10*, *ABCA6*, *ABCA8*, *ABCA9*, *ABCG2*, *ABHD12B*, *ABI3BP*, *ABLIM1*, *ACAD9*, *ADIPOQ*, *AFF1*, *AFF3*, *AGBL3*, *ANK2*, *AQP7*, *ARFGEF3*, *ARHGAP10*, *ATRNL1*, *BST1*, *BTBD8*, *CAB39L*, *CAMK2D*, *CAST*, *CCDC141*, *CDH13*, *CEP128*, *CPEB2*, *CPED1*, *CSRP3*, *CYP21A2*, *DACH1*, *DENND2A*, *DENND4A*, *DEPTOR*, *DLC1*, *DOCK9*, *DPYSL2*, *DSC1*, *EBF2*, *EBF3*, *EGFR*, *ELL2*, *EMCN*, *ENTPD1*, *ERG*, *ERICH3*, *ESR1*, *F13A1*, *FABP4*, *FAM214A*, *FAT4*, *FAXDC2*, *FHL1*, *FLVCR2*, *GALNT15*, *GALNT18*, *GHR*, *HIF3A*, *HMCN2*, *IL33*, *INPP4B*, *JAM2*, *KDM7A*, *KLHDC8A*, *KLHL2*, *LAMA2*, *LAMA3*, *LDB2*, *LDB3*, *LMOD1*, *LNPEP*, *LONP2*, *LRP2BP*, *LTBP4*, *LVRN*, *MAN1A1*, *MAN1C1*, *MAOB*, *MAP3K20*, *ME3*, *MEF2C*, *MINDY3*, *MLXIPL*, *MMRN1*, *MOV10L1*, *MOXD1*, *MYH11*, *MYRIP*, *NFKB1*, *NOSTRIN*, *NOVA1*, *NWD2*, *PCK1*, *PDGFD*, *PDK4*, *PEBP4*, *PIK3C2G*, *PITPNC1*, *PLD1*, *PLPP1*, *PPARG*, *PPP1R3A*, *PRDM5*, *PREX2*, *PROX1*, *PYGM*, *RASGEF1B*, *RBMS2*, *RCL1*, *RERGL*, *RFX2*, *RHOJ*, *RORA*, *RPS6KA5*, *SCARA5*, *SCN7A*, *SETBP1*, *SH3D19*, *SIK2*, *SLC28A3*, *SLC36A4*, *SMAD1*, *SNCAIP*, *SNED1*, *SYBU*, *SYNE1*, *TGFBR3*, *TLL1*, *TMOD1*, *TMTC1*, *TNS1*, *USP53*, *UST*, *VIPR2*, *ZBTB16*, *ZMAT1*, *ZNF704* |
| Gene symbols not available replaced with ensembl ID. Data based on based on average of logarithmic scale base two ($log_2$). |

**Supplementary table 4.** Top DEGs across all sample groups.

| Gene symbol | Ensembl ID | CLs | LB *f*HER2- | Normal-like TN | Basal-like TN |
|---|---|---|---|---|---|
| ***Top upregulated DEGs**** | | | | | |
| *ASNS* | ENSFCAG00000022830 | top↑ | - | - | ↑ |
| *CDCA3* | ENSFCAG00000004274 | ↑ | ↑ | ↑ | top↑ |
| *DDIAS* | ENSFCAG00000031353 | ↑ | top↑ | ↑ | ↑ |
| *DMBT1* | ENSFCAG00000003657 | ↓ | - | top↑ | - |
| *FADS2* | ENSFCAG00000024030 | ↑ | ↑ | top↑ | ↑ |
| *FKBP10* | ENSFCAG00000002137 | ↑ | top↑ | ↑ | ↑ |
| *HSPH1* | ENSFCAG00000007547 | ↑ | ↑ | ↑ | top↑ |
| *MMP9* | ENSFCAG00000014014 | ↓ | top↑ | ↑ | ↑ |
| *MMP11* | ENSFCAG00000015309 | - | top↑ | ↑ | ↑ |
| *NEK2* | ENSFCAG00000023718 | ↑ | ↑ | top↑ | top↑ |
| *OTUD3* | ENSFCAG00000028330 | top↑ | - | - | - |
| *RACGAP1* | ENSFCAG00000009459 | top↑ | top↑ | top↑ | top↑ |
| *TF* | ENSFCAG00000009592 | top↑ | - | ↑ | ↑ |
| *TK1* | ENSFCAG00000013007 | ↑ | ↑ | top↑ | ↑ |
| *VLDLR* | ENSFCAG00000013257 | ↑ | - | ↑ | top↑ |
| *ZNF318* | ENSFCAG00000009726 | top↑ | - | - | ↑ |
| ***Top downregulated DEGs**** | | | | | |
| *ABCA8* | ENSFCAG00000006139 | ↓ | ↓ | top↓ | ↓ |
| *ABCA10* | ENSFCAG00000030918 | ↓ | ↓ | top↓ | ↓ |
| *ABLIM1* | ENSFCAG00000000635 | top↓ | top↓ | ↓ | ↓ |

| | | | | | |
|---|---|---|---|---|---|
| *DPP4* | ENSFCAG00000004460 | ↓ | ↓ | top↓ | ↓ |
| *EGFR* | ENSFCAG00000015468 | top↓ | ↓ | ↓ | ↓ |
| *ERICH3* | ENSFCAG00000031192 | ↓ | top↓ | ↓ | ↓ |
| *GHR* | ENSFCAG00000026499 | ↓ | ↓ | top↓ | top↓ |
| *HIF3A* | ENSFCAG00000014181 | ↓ | ↓ | ↓ | top↓ |
| *MOV10L1* | ENSFCAG00000011907 | ↓ | top↓ | ↓ | ↓ |
| *NIBAN1* | ENSFCAG00000025716 | top↓ | - | - | ↓ |
| *NOSTRIN* | ENSFCAG00000010646 | ↓ | ↓ | ↓ | top↓ |
| *PDK4* | ENSFCAG00000028642 | ↓ | top↓ | ↓ | ↓ |
| *PTCH2* | ENSFCAG00000009971 | ↓ | - | - | top↓ |
| *RASGEF1B* | ENSFCAG00000028077 | top↓ | ↓ | ↓ | ↓ |
| *RERGL* | ENSFCAG00000005941 | ↓ | top↓ | ↓ | top↓ |
| *SCARA5* | ENSFCAG00000031961 | ↓ | ↓ | top↓ | ↓ |
| *TXNIP* | ENSFCAG00000029919 | top↓ | - | - | - |

*Top: highest significance within sample group. ↑ upregulated, ↓ downregulated, and – not differentially expressed.

**Supplementary table 5.** Recurrent DEGs across all sample types associated with selected KEGG pathways.

| Category | Selected pathways (KEGG code) | Genes | FDR |
|---|---|---|---|
| Cell growth and death | Cell cycle (fca04110) | ***BUB1***, ***BUB1B***, ***CCNB1***, ***CCNB2***, ***CDC25B***, ***CDC6***, ***CDK1***, ***CDK4***, ***CHEK1***, ***E2F2***, ***ESPL1***, ***MCM4***, ***MCM5***, ***ORC6***, ***PCNA***, ***PKMYT1***, ***PTTG1***, ***TTK*** | 2.80E-12** |
| | p53 signalling pathway (fca04115) | ***CCNB1***, ***CCNB2***, ***CDK1***, ***CDK4***, ***CHEK1***, ***GTSE1***, ***RRM2*** | 2.95E-03** |
| Replication and repair | DNA replication (fca03030) | ***FEN1***, ***MCM4***, ***MCM5***, ***PCNA***, ***POLD1*** | 1.24E-02** |
| Metabolic-related | Glycolysis / Gluconeogenesis (fca00010) | ***AKR1A1***, ***ENO1***, *PCK1*, ***PCK2***, ***PFKL***, ***PFKP***, ***PGK1***, ***PKM*** | 1.59E-02*<br>2.95E-03** |
| | Central carbon metabolism in cancer (fca05230) | ***PFKL***, ***PFKP***, ***PKM***, ***SLC16A3***, ***SLC1A5***, ***SLC2A1*** | 1.24E-02** |
| | Biosynthesis of amino acids (fca01230) | ***ENO1***, ***PFKL***, ***PFKP***, ***PGK1***, ***PKM***, ***SHMT2*** | 1.77E-02** |
| Endocrine-related | Progesterone-mediated oocyte maturation (fca04914) | ***BUB1***, ***CCNB1***, ***CCNB2***, ***CDC25B***, ***CDK1***, ***PKMYT1*** | 3.96E-02** |
| Protein processing | Protein processing in endoplasmic reticulum (fca04141) | ***CALR***, ***HSP90B1***, ***HSPA5***, ***HSPA8***, ***HSPH1***, ***HYOU1***, ***MOGS***, ***PDIA4***, ***PDIA6***, ***PRKCSH*** | 2.95E-03** |
| Cancer-related | HTLV-I infection (fca05166) | ***BUB1B***, ***CALR***, ***CDK4***, ***CHEK1***, ***E2F2***, ***KAT2A***, ***LTBR***, ***PCNA***, ***POLD1***, ***PTTG1***, ***SLC25A5***, ***SLC2A1*** | 2.95E-03** |

KEGG, Kyoto Encyclopedia of Genes and Genomes; and DEGs, differentially expressed genes. Gene symbols not available replaced with ensembl ID. DEGs in analysis: 340, up-regulated genes (n = 203), down-regulated genes (n = 137). Up-regulated DEGs in bold, and down-regulated DEGs in non-bold. *FDR for total DEGs (up- and down-regulated DEGs together), and **FDR for up-regulated DEGs. Data based on average of logarithmic scale base two ($log_2$). Data analysed with DAVID (https://david.ncifcrf.gov/).

**Supplementary table 6.** Differentially expressed genes in basal-like triple negative tumours associated with selected KEGG pathways.

| Category | Selected pathway (KEGG code) | Gene symbol | FDR |
|---|---|---|---|
| Adhesion, and cell interaction | ECM-receptor interaction (fca04512) | *CD36*, ***COL1A1***, ***COL1A2***, ***COL2A1***, *COL5A3*, ***COMP***, ***HMMR***, *ITGA6*, ***FN1***, *LAMA2*, *LAMA3*, *RELN*, ***SDC1***, ***SDC4***, ***TNC***, ***TNN***, *VTN* | 2.25E-02* |
| | Focal adhesion (fca04510) | *ACTN3*, *AKT3*, *COL5A3*, *EGFR*, *FYN*, *IGF1*, *ITGA5*, *LAMA2*, *LAMA3*, *LAMA4*, *LAMC1*, *PDGFC*, *PDGFD*, *PTEN*, *SOS2*, *VCL*, *VEGFC*, *VTN* | 4.94E-02*** |
| Cell growth and death | Cell cycle (fca04110) | ***BUB1***, ***BUB1B***, ***CCNB1***, ***CCNB2***, ***CDC25A***, ***CDC25B***, ***CDC6***, ***CDK1***, ***CDK4***, ***CDKN2A***, ***CHEK1***, ***E2F2***, ***ESPL1***, ***HDAC1***, ***MAD2L1***, ***MCM4***, ***MCM5***, ***ORC6***, ***PCNA***, ***PKMYT1***, ***PTTG1***, *RB1*, ***TTK***, ***YWHAB*** | 2.23E-03*<br>2.05E-06** |
| | p53 signalling pathway (fca04115) | ***CCNB1, CCNB2, CDK1, CDK4, CDKN2A, CHEK1, GTSE1, RRM2, RRM2B,*** *SESN1,* ***SESN2*** | NS |
| Metabolic-related | Metabolic pathways (fca01100) | *AADAT*, *AASS*, ***ACAA1***, *AGPAT4*, ***AK2***, ***AKR1A1***, ***ALDH1A3***, ***ALG3***, *ASNS*, ***ASPA***, ***ATP5F1C***, ***AZIN2***, ***B3GNT4***, ***B4GALNT1***, ***B4GALT4***, ***BPNT1***, *BST1*, ***CDO1***, *CKM*, ***CKMT1A***, ***COX4I2***, *CSGALNACT1*, ***CYC1***, *CYP21A2*, ***CYP26A1***, *CYP27A1*, ***CYP27B1***, ***CYP51A1***, ***DEGS1***, ***DGAT1***, ***DHCR24***, ***DHCR7***, *DPYS*, ***ECHS1***, ***ENO1***, ***ENSFCAG00000025259***, *ENSFCAG00000030205*, ***FAH***, *FAXDC2*, ***FLAD1***, ***GALE***, ***GALK1***, ***GALNT12***, *GALNT15*, ***GALNT6***, ***GCNT1***, ***GCNT3***, ***GOT1***, ***GOT2***, ***GPAA1***, *GPAT3*, *HDC*, *HGSNAT*, ***HK2***, ***HMBS***, *HPGDS*, ***HPRT1***, ***HSD17B7***, *JMJD7*, ***LAP3***, ***LIPC***, *LPIN1*, *MAN1C1*, *MAOB*, ***MDH1***, ***MDH2***, *ME3*, ***MOGS***, ***MPI***, ***MTHFD2***, *MTR*, ***MVK***, ***P4HA2***, *PCK1*, ***PCK2***, ***PDHB***, ***PFKL***, ***PFKP***, *PGAP1*, ***PGD***, ***PGK1***, ***PHGDH***, ***PIGQ***, ***PIGT***, *PIK3C2G*, ***PKM***, ***PLA2G3***, *PLA2G4E*, ***PLA2G7***, ***PLD4***, *PLPP1*, ***POLD1***, ***POLR2E***, ***PSAT1***, ***PTGES2***, *PYGM*, ***RPN2***, ***RRM2***, ***RRM2B***, *SGMS1*, ***SHMT2***, *SMPD3*, ***SPTLC1***, ***SQLE***, ***ST3GAL4***, *SUCLG2*, ***TK1***, ***TST***, ***UMPS*** | 2.25E-02*<br>2.70E-03** |
| | Carbon metabolism (fca01200) | ***ECHS1***, ***ENO1***, ***GOT1***, ***GOT2***, ***HK2***, ***MDH1***, ***MDH2***, ***PDHB***, ***PFKL***, ***PFKP***, ***PGD***, ***PGK1***, ***PHGDH***, ***PKM***, ***PSAT1***, ***SHMT2*** | 2.70E-03** |
| | Glycolysis / Gluconeogenesis (fca00010) | ***AKR1A1***, ***ALDH1A3***, ***ENO1***, ***HK2***, ***PCK2***, ***PDHB***, ***PFKL***, ***PFKP***, ***PGK1***, ***PKM*** | 3.00E-02** |
| | Steroid biosynthesis (fca00100) | ***CYP27B1***, ***CYP51A1***, ***DHCR24***, ***DHCR7***, ***HSD17B7***, ***SQLE*** | 3.97E-02** |
| Signal transduction | PI3K-Akt signalling pathway (fca04151) | ***BRCA1,*** *EGFR, COL5A3,* ***TNN, HSP90B1****, VTN,* ***DDIT4, VEGFA, PCK2, TLR2, STK11****, PRLR, ANGPT4,* ***COL1A1, OSM****, GHR,* ***COL1A2, COMP****, VEGFC,* ***FN1****, NFKB1, IGF1R, LAMA2, PIK3R1, YWHAB,* ***COL2A1, CDK4****, FGF7, TEK, ITGA6, FLT4, PDGFD, LAMA3,* ***PDGFB****, RELN,* ***TNC****, PCK1,* ***HSP90AA1*** | NS |
| Protein processing | Protein processing in endoplasmic reticulum (fca04141) | ***CALR***, ***DNAJB11***, ***ERP29***, ***HSP90AA1***, ***HSP90B1***, ***HSPA1L***, ***HSPA2***, ***HSPA5***, ***HSPA8***, ***HSPH1***, ***HYOU1***, ***MOGS***, ***NSFL1C***, ***PDIA3***, ***PDIA4***, ***PDIA6***, ***PRKCSH***, ***RPN2***, ***RRBP1***, ***SEC62***, ***SSR4*** | 1.44E-03** |

| | | | |
|---|---|---|---|
| Immune-related | Antigen processing and presentation (fca04612) | ***CALR***, ***CTSB***, ***ENSFCAG00000009202***, ***ENSFCAG00000009442***, ***HSP90AA1***, ***HSPA1L***, ***HSPA2***, ***HSPA8***, ***IFI30***, ***PDIA3*** | 2.71E-02** |

KEGG, Kyoto Encyclopedia of Genes and Genomes; and DEGs, differentially expressed genes. Gene symbols not available replaced with ensembl ID. DEGs in analysis: 1279, up-regulated genes (n = 795), down-regulated genes (n = 484), exclusively up-regulated genes (n = 102), and exclusively down-regulated genes (n = 31). Up-regulated DEGs in bold, down-regulated DEGs in non-bold, exclusive DEGs are underlined. NS, not significant, *FDR for total DEGs (up- and down-regulated together), **FDR for up-regulated DEGs, and ***FDR for down-regulated DEGs. FDR for exclusive DEGs (up- or downregulated) are underlined. Data based on average of logarithmic scale base two ($log_2$). Data analysed with DAVID (https://david.ncifcrf.gov/).

**Supplementary table 7.** Differentially expressed genes in normal-like triple negative tumours associated with selected KEGG pathways.

| **Category** | **Term** | **Genes** | FDR |
|---|---|---|---|
| Adhesion, and cell interaction | ECM-receptor interaction (fca04512) | *CD36*, *CD47*, ***COL2A1***, ***COL11A2***, *COL5A3*, ***HMMR***, *ITGA5*, ***ITGB4***, ***FN1***, *LAMA2*, *LAMA3*, *LAMA4*, ***LAMA5***, *LAMC1*, ***SDC1***, ***SDC4***, ***SV2B***, ***SV2C***, ***TNN***, *VTN* | 1.10E-02* |
| Membrane transport | ABC transporters (fca02010) | *ABCA1*, ***ABCA13***, ***ABCA3***, *ABCA6*, *ABCA8*, *ABCA9*, ***ABCB11***, ***ABCB5***, ***ABCB6***, ***ABCB9***, ***ABCC12***, ***ABCC8***, ***ABCD1***, *ABCG2* | 4.30E-02* |
| Cell growth and death | Cell cycle (fca04110) | ***ANAPC11***, ***BUB1***, ***BUB1B***, ***CCNB1***, ***CCNB2***, ***CDC25A***, ***CDC25B***, ***CDC25C***, ***CDC45***, ***CDC6***, ***CDK1***, ***CDK4***, ***CHEK1***, ***E2F2***, ***ESPL1***, ***FZR1***, ***HDAC1***, ***MCM2***, ***MCM3***, ***MCM4***, ***MCM5***, ***MCM6***, ***MCM7***, ***ORC1***, ***ORC6***, ***PCNA***, ***PKMYT1***, ***PLK1***, ***PTTG1***, *RB1*, ***TP53***, ***TTK***, ***WEE2*** | 2.10E-03*<br>1.45E-06** |
| | p53 signalling pathway (fca04115) | ***BAX, CCNB1, CCNB2, CDK1, CDK4, CHEK1, GTSE1,*** *IGF1, PTEN,* ***RRM2, RRM2B,*** *SESN1*, ***TP53*** | 4.55E-02* |
| Metabolic-related | Metabolic pathways (fca01100) | *ACACB*, *ACSBG1*, *ACSL4*, ***ACSL6***, ***ACSM1***, ***ACSM5***, ***ADO***, *AGPAT4*, ***AHCY***, ***AK5***, ***AKR1A1***, ***AKR1D1***, ***ALAS1***, *ALAS2*, ***ALDH1A3***, ***ALDH5A1***, ***ALG12***, *ALOX12B*, ***APIP***, ***ATP5F1A***, ***B3GNT4***, ***B4GALNT1***, ***B4GALT2***, ***B4GALT3***, ***BCO1***, *BST1*, ***C1GALT1C1***, ***CAD***, ***CDO1***, *CKM*, ***CKMT1A***, ***COQ7***, ***CPS1***, *CSGALNACT2*, ***CYC1***, ***CYP19A1***, *CYP21A2*, ***CYP26A1***, ***DAO***, ***DBH***, ***DCK***, ***DCT***, ***DDC***, ***DEGS1***, ***DGAT1***, ***DGKK***, ***DGKQ***, ***DHCR24***, ***DHCR7***, ***ENO1***, ***ENO2***, *ENSFCAG00000010631*, ***ENSFCAG00000011675***, *ENSFCAG00000023472*, ***ENSFCAG00000024210***, ***ENSFCAG00000024226***, ***ENSFCAG00000025259***, ***EXTL1***, ***FAH***, ***FAHD1***, *FAXDC2*, ***FLAD1***, ***FTCD***, ***GAD1***, ***GALE***, ***GALK1***, ***GALNS***, ***GALNT12***, ***GALNT14***, *GALNT15*, ***GCNT1***, *GCNT2*, ***GCNT3***, *GFPT2*, *GK*, ***GLCE***, *GLS*, *GNE*, ***GPAA1***, ***GPI***, ***H6PD***, ***HAO1***, ***HAO2***, ***HCN3***, ***HMBS***, ***HPRT1***, ***HSD17B3***, ***HYAL3***, ***IMPDH2***, ***INPP5E***, ***ITPKA***, ***KMO***, ***LIPC***, ***LIPF***, *LPIN1*, *MAN1C1*, *MAN2A1*, ***MAN2A2***, *MAOB*, ***MCAT***, *ME3*, ***MOGS***, ***MTHFD2***, ***MVD***, ***NAGS***, *NAMPT*, ***NME5***, *NT5C2*, ***OGDHL***, ***OTC***, ***PAFAH1B3***, ***PAH***, *PANK3*, ***PAPSS2***, ***PC***, *PCK1*, ***PCK2***, ***PCYT1B***, ***PEMT***, ***PFAS***, ***PFKL***, ***PFKP***, ***PGD***, ***PGK1***, ***PGS1***, ***PHGDH***, ***PIGQ***, ***PIGT***, *PIK3C2G*, ***PIPOX***, ***PKM***, *PLA2G4E*, ***PLA2G7***, ***PLCB3***, ***PLCZ1***, ***PLD2***, ***PLD4***, *PLPP1*, ***PLPP2***, ***PNLIPRP1***, *PNMT*, *PNPLA2*, ***POLD1***, ***POLE***, ***POLR3C***, ***PRIM1***, ***PSAT1***, ***PTGES2***, *PTGS1*, *PYGM*, ***QARS1***, ***RRM2***, ***RRM2B***, | 7.33E-03*<br>1.51E-04** |

| | | | |
|---|---|---|---|
| | | ***SARDH***, ***SHMT2***, *SMPD3*, ***SPAM1***, *SPTLC2*, ***ST3GAL4***, ***ST3GAL6***, ***ST8SIA5***, *SUCLG2*, *SYNJ1*, ***TK1***, ***TKT***, ***TKTL1***, ***TST***, ***TYMS***, ***TYRP1***, *UAP1*, ***UGT1A1***, ***UGT2A2***, ***UGT8***, ***UPB1***, ***UQCRC1***, ***UROC1*** | |
| | Biosynthesis of amino acids (fca01230) | ***CPS1***, ***ENO1***, ***ENO2***, ***ENSFCAG00000024226***, ***HCN3***, ***NAGS***, ***OTC***, ***PAH***, ***PC***, ***PFKL***, ***PFKP***, ***PGK1***, ***PHGDH***, ***PKM***, ***PSAT1***, ***SHMT2***, ***TKT***, ***TKTL1*** | 1.11E-03** |
| | Carbon metabolism (fca01200) | ***CPS1***, ***ENO1***, ***ENO2***, ***GPI***, ***H6PD***, ***HAO1***, ***HAO2***, ***HCN3***, ***OGDHL***, ***PC***, ***PFKL***, ***PFKP***, ***PGD***, ***PGK1***, ***PHGDH***, ***PKM***, ***PSAT1***, ***SHMT2***, ***TKT***, ***TKTL1*** | 3.73E-02** |
| Environmental Information Processing | Neuroactive ligand-receptor interaction (fca04080) | ***ADORA1***, ***ADORA3***, ***ADRA1D***, ***CALCR***, ***CHRM4***, ***CHRNA6***, ***CRHR1***, ***DRD3***, ***ENSFCAG00000018949***, ***FSHR***, ***GABRA1***, ***GABRA3***, ***GABRB2***, ***GABRB3***, ***GABRP***, ***GABRQ***, ***GABRR1***, *GHR*, ***GLP1R***, ***GLP2R***, ***GLRA1***, ***GLRA2***, ***GLRA3***, ***GRIA1***, ***GRIA2***, ***GRIK2***, ***GRIK3***, ***GRIN2A***, ***GRIN2D***, ***GRM3***, ***GRM6***, ***GRM8***, *GRPR*, ***HCRTR2***, ***HRH4***, *HTR2B*, ***LHCGR***, *LPAR1*, ***LPAR2***, ***MCHR2***, ***NMBR***, ***NPFFR2***, ***OPRK1***, ***OPRM1***, *PARD3*, ***PLG***, *PRLR*, *PTH1R*, ***SCTR***, ***TACR2***, *THRB*, ***TSHR***, *VIPR2* | 7.33E-03*<br>3.48E-04**<br>1.98E-06** |
| Replication and repair | DNA replication (fca03030) | ***DNA2***, ***FEN1***, ***LIG1***, ***MCM2***, ***MCM3***, ***MCM4***, ***MCM5***, ***MCM6***, ***MCM7***, ***PCNA***, ***POLD1***, ***POLE***, ***PRIM1***, ***RNASEH2A*** | 1.51E-04** |
| Adhesion, and cell interaction | Focal adhesion (fca04510) | *ACTN3*, *AKT3*, *COL5A3*, *EGFR*, *FYN*, *IGF1*, *ITGA5*, *LAMA2*, *LAMA3*, *LAMA4*, *LAMC1*, *PDGFC*, *PDGFD*, *PTEN*, *SOS2*, *VCL*, *VEGFC*, *VTN* | 2.61E-02*** |
| Signal transduction | PI3K-Akt signalling pathway (fca04151) | *AKT3*, *ATF2*, *COL5A3*, *CREB1*, *CREB3L1*, *CREB5*, *EGFR*, *GHR*, *IGF1*, *IL7R*, *ITGA5*, *JAK1*, *JAK2*, *LAMA2*, *LAMA3*, *LAMA4*, *LAMC1*, *LPAR1*, *NFKB1*, *PCK1*, *PDGFC*, *PDGFD*, *PPP2R2A*, *PRLR*, *PTEN*, *SOS2*, *VEGFC*, *VTN* | 7.73E-03*** |
| | MAPK signalling pathway (fca04010) | *AKT3*, *ATF2*, *CACNA1C*, *CACNA2D1*, *DUSP16*, *EGFR*, *MAP3K1*, *MAP3K20*, *MAPK7*, *MEF2C*, *NFATC1*, *NFKB1*, *PLA2G4E*, *PPP3CA*, *PPP3CC*, *RAPGEF2*, *RPS6KA3*, *RPS6KA5*, *RRAS2*, *SOS2*, *TAOK3*, *TGFBR2* | 7.73E-03*** |
| Endocrine-related | Insulin resistance (fca04931) | *ACACB*, *AKT3*, *CD36*, *CREB1*, *CREB3L1*, *CREB5*, *GFPT2*, *MLXIPL*, *NFKB1*, *OGT*, *PCK1*, *PPP1R3A*, *PTEN*, *PYGM*, *RPS6KA3* | 6.93E-03*** |
| | Glucagon signalling pathway (fca04922) | *ACACB*, *AKT3*, *ATF2*, *CAMK2D*, *CREB1*, *CREB3L1*, *CREB5*, *PCK1*, *PPP3CA*, *PPP3CC*, *PYGM*, *SIK1*, *SIK2* | 7.73E-03*** |
| | PPAR signalling pathway (fca03320) | *ACSBG1*, *ACSL4*, *ADIPOQ*, *AQP7*, *CD36*, *FABP4*, *GK*, *LPL*, *PCK1*, *PPARG*, *SORBS1* | 7.73E-03*** |
| Cancer-related | Prostate cancer (fca05215) | *AKT3*, *CREB1*, *CREB3L1*, *CREB5*, *EGFR*, *IGF1*, *NFKB1*, *PDGFC*, *PDGFD*, *PTEN*, *RB1*, *SOS2* | 1.00E-02*** |
| Development and regeneration | Osteoclast differentiation (fca04380) | *AKT3*, *CREB1*, *FCGR2B*, *FOSB*, *FYN*, *GAB2*, *JAK1*, *MITF*, *NFATC1*, *NFKB1*, *PPARG*, *PPP3CA*, *PPP3CC*, *TGFBR2* | 1.12E-02*** |

KEGG, Kyoto Encyclopedia of Genes and Genomes; and DEGs, differentially expressed genes. Gene symbols not available replaced with ensembl ID. DEGs in analysis: 2406, up-regulated genes (n = 1772), down-regulated genes (n = 634), exclusively up-regulated genes (n = 691), and exclusively down-regulated genes (n = 100). Up-regulated DEGs in bold, down-regulated DEGs in non-bold, exclusive DEGs are underlined. *FDR for total DEGs (up- and down-regulated together), **FDR for up-regulated DEGs, and ***FDR for down-regulated DEGs. FDR

for exclusive DEGs (up- or downregulated) are underlined. Data based on average of logarithmic scale base two ($log_2$). Data analysed with DAVID (https://david.ncifcrf.gov/).

**Supplementary table 8.** Differentially expressed genes in Luminal B *f*HER2 negative tumours associated with selected KEGG pathways and GO terms.

| **Category** | **Selected pathway (KEGG code)** | **Genes** | **FDR** |
|---|---|---|---|
| Signal transduction | PI3K-Akt signalling pathway (fca04151) | *AKT3*, ***CDK2***, ***CDK4***, ***COL1A1***, ***COL1A2***, ***COL2A1***, ***COL3A1***, ***COL4A1***, ***COL4A2***, ***COL5A2***, ***COL6A2***, ***COL6A3***, ***CSF1***, ***CSF1R***, ***DDIT4***, *EGF*, *EGFR*, ***FGFR2***, ***FN1***, *GHR*, ***HSP90B1***, *INSR*, ***ITGA10***, *ITGA8*, ***ITGAV***, ***ITGB1***, ***JAK3***, *LAMA2*, *LAMA3*, ***LAMB1***, *LAMB4*, *NFKB1*, ***OSM***, *PCK1*, ***PCK2***, *PDGFD*, *RELN*, *TEK*, ***THBS3***, ***TNC***, ***TNN***, ***TP53***, ***YWHAB*** | 3.37E-02* |
| Endocrine-related | Progesterone-mediated oocyte maturation (fca04914) | *ADCY2*, *ADCY5*, *ADCY9*, *AKT3*, ***BUB1***, ***CCNA2***, ***CCNB1***, ***CCNB2***, ***CDC25B***, ***CDK1***, ***CDK2***, *CPEB1*, *CPEB4*, ***MAD2L1***, ***PKMYT1***, ***PLK1*** | 3.93E-02* |
| | Insulin resistance (fca04931) | *ACACB*, *AKT3*, *GFPT2*, *INSR*, *MLXIPL*, *NFKB1*, *PCK1*, *PPP1R3A*, *PRKAG3*, *PRKCQ*, *PYGM*, *SLC27A6* | 1.84E-02*** |
| Cancer-related | Small cell lung cancer (fca05222) | *AKT3*, ***CDK2***, ***CDK4***, ***COL4A1***, ***COL4A2***, ***E2F2***, ***FN1***, ***ITGAV***, ***ITGB1***, *LAMA2*, *LAMA3*, ***LAMB1***, *LAMB4*, *NFKB1*, ***TP53***, ***TRAF2*** | 3.37E-02* |
| | HTLV-I infection (fca05166) | ***BUB1B***, ***CALR***, ***CANX***, ***CDK4***, ***CHEK1***, ***E2F2***, ***ENSFCAG00000000629***, ***ICAM1***, ***ITGB2***, ***JAK3***, ***KAT2A***, ***LTBR***, ***MAD2L1***, ***PCNA***, ***POLD1***, ***POLE***, ***PTTG1***, ***RAN***, ***SLC25A5***, ***SLC2A1***, ***SMAD3***, ***SPI1***, ***TGFB1***, ***TP53***, ***VDAC1***, ***VDAC2*** | 1.44E-02** |
| Development and regeneration | Osteoclast differentiation (fca04380) | *AKT3*, *CAMK4*, ***CSF1***, ***CSF1R***, ***FCGR3***, ***FCGR3A***, ***FHL2***, *GAB2*, *MITF*, ***NCF4***, *NFKB1*, *PPARG*, ***SPI1***, ***STAT1***, ***STAT2***, ***TGFB1***, ***TNFSF11***, ***TRAF2***, ***TREM2***, ***TYROBP*** | 3.97E-02* |
| Metabolic-related | Glycolysis / Gluconeogenesis (fca00010) | ***AKR1A1***, ***ALDH3B1***, ***DLAT***, ***ENO1***, *FBP1*, *GAPDHS*, ***HK1***, ***LDHA***, *PCK1*, ***PCK2***, ***PFKL***, ***PFKP***, ***PGK1***, ***PKM*** | 1.99E-02*<br>1.44E-02** |
| | N-Glycan biosynthesis (fca00510) | ***ALG3***, ***B4GALT2***, ***DDOST***, ***DPAGT1***, *MAN1C1*, ***MGAT1***, ***MGAT2***, ***MOGS***, ***RPN1***, ***RPN2***, ***STT3A*** | 4.66E-02*<br>1.31E-02** |
| Adhesion, and cell interaction | ECM-receptor interaction (fca04512) | ***COL1A1***, ***COL1A2***, ***COL2A1***, ***COL3A1***, ***COL4A1***, ***COL4A2***, ***COL5A2***, ***COL6A2***, ***COL6A3***, ***HMMR***, ***HSPG2***, ***ITGA10***, *ITGA8*, ***ITGAV***, ***ITGB1***, ***FN1***, *LAMA2*, *LAMA3*, ***LAMB1***, *LAMB4*, *RELN*, ***SDC1***, ***THBS3***, ***TNC***, ***TNN*** | 2.06E-06*<br>4.69E-06** |
| | Focal adhesion (fca04510) | ***ACTN1***, *ACTN2*, *AKT3*, ***BCAR1***, ***COL1A1***, ***COL1A2***, ***COL2A1***, ***COL3A1***, ***COL4A1***, ***COL4A2***, ***COL5A2***, ***COL6A2***, ***COL6A3***, *EGF*, *EGFR*, ***FLNA***, ***FN1***, ***ITGA10***, *ITGA8*, ***ITGAV***, ***ITGB1***, *LAMA2*, *LAMA3*, ***LAMB1***, *LAMB4*, ***PARVB***, *PDGFD*, ***RAC2***, *RELN*, ***SRC***, ***THBS3***, ***TNC***, ***TNN***, ***VASP***, *VAV3* | 9.42E-04*<br>8.35E-03** |
| Protein processing | Protein processing in endoplasmic reticulum (fca04141) | ***BMP6***, ***CALR***, ***CANX***, ***DDOST***, ***DNAJB11***, ***ERP29***, ***HSP90B1***, ***HSPA5***, ***HSPA8***, ***HSPH1***, ***HYOU1***, *MAN1C1*, ***MOGS***, ***OS9***, ***P4HB***, ***PDIA3***, ***PDIA4***, ***PDIA6***, ***PRKCSH***, ***RPN1***, ***RPN2***, ***RRBP1***, ***SEC61A1***, ***SSR1***, ***SSR3***, ***STT3A***, ***TRAF2*** | 1.19E-02*<br>2.95E-05** |

| | | | |
|---|---|---|---|
| Cell growth and death | Cell cycle (fca04110) | ***BUB1, BUB1B, CCNA2, CCNB1, CCNB2, CDC25B, CDC45, CDC6, CDK1, CDK2, CDK4, CHEK1, E2F2, ESPL1, MAD2L1, MCM2, MCM3, MCM4, MCM5, MCM6, ORC1, ORC6, PCNA, PKMYT1, PLK1, PTTG1, SMAD3, TFDP1, TGFB1, TP53, TTK, YWHAB*** | 5.18E-12** |
| | p53 signalling pathway (fca04115) | ***CCNB1, CCNB2, CD82, CDK1, CDK2, CDK4, CHEK1, GTSE1, RRM2, SERPINE1, TP53*** | 3.19E-02** |
| Replication and repair | DNA replication (fca03030) | ***FEN1, MCM2, MCM3, MCM4, MCM5, MCM6, PCNA, POLD1, POLE, RPA1*** | 1.86E-03** |
| Transport and catabolism | Phagosome (fca04145) | ***ATP6V1A, ATP6V1B2, ATP6V1E1, ATP6V1F, CALR, CANX, CLEC7A, ENSFCAG00000000629, FCGR3, FCGR3A, ITGAV, ITGB1, ITGB2, M6PR, MRC2, NCF4, PDP, RAB5C, SEC61A1, TCIRG1, TFRC, THBS3, TUBB*** | 4.83E-05**<br>2.15E-03** |
| | Lysosome (fca04142) | ***AP3B2, CTSB, CTSZ, GM2A, GNPTAB, GUSB, HEXB, M6PR, MAN2B1, NAGA, NEU1, NPC2, PDP, PPT1, PSAP, SLC11A1, TCIRG1*** | 8.35E-03**<br>2.57E-03** |
| Extracellular exosome | Extracellular exosome (GO:0070062) | ***A2M, AARS1, ABHD8, ACTN1, AEBP1, AHCY, AK2, AKR1A1, ALDH16A1, ALDH1L2, ALDH3B1, ANPEP, ANXA6, ARPC4, ARPC5L, ATAD2, ATP5F1C, ATP6V1A, ATP6V1B2, ATP6V1E1, ATP6V1F, BMP6, C1QA, C1QB, C1S, CAD, CALR, CANX, CCT2, CCT5, CCT7, CCT8, CD248, CD70, CD82, CDH11, CDK1, CFL1, CFL2, CILP2, CKB, CLIC1, CLMP, CLSTN3, CNP, COL1A2, COL4A2, COL6A2, COL6A3, CSF1, CSK, CSPG4, CTSB, CTSZ, DNASE1L1, DNPH1, DPP3, EFNB1, ENO1, ENSFCAG00000000179, ENSFCAG00000011849, ENSFCAG00000022771, ERP29, FERMT3, FKBP4, FLNA, FLOT1, FN1, FUCA1, GALE, GDF15, GGH, GM2A, GPC4, GRN, GUSB, HEXB, HSP90B1, HSPA5, HSPG2, HSPH1, HTRA1, HYOU1, ICAM1, ICAM3, ISLR, ITGAV, ITGB1, ITGB2, KIRREL1, LAMB1, LAMTOR1, LRRC15, LUM, MAPKAPK2, MARS1, MGAT1, MMP9, MOGS, MVP, MXRA5, MYH9, MYO1G, NAGA, NCS1, NEU1, NPC2, NUTF2, P3H1, P4HB, PA2G4, PCK2, PCNA, PCOLCE, PDIA3, PDIA6, PFKL, PFKP, PLAT, PLAU, PLAUR, PLBD2, PLOD1, PLOD3, POGLUT1, PPT1, PRDX4, PSAP, PSMB3, PSMD13, PSMD2, PSMD3, PSMD6, PTGS1, QSOX1, RAB5C, RAC2, RACGAP1, RALA, RAN, RAP2C, RHOG, RRM1, RUVBL1, RUVBL2, S100A8, SDC1, SERPINE1, SERPINF1, SERPINF2, SERPINH1, SHMT2, SLC1A4, SLC1A5, SLC2A1, SLC35F6, SLC37A2, SMC2, SMPDL3A, SRC, SRPRA, ST3GAL4, STEAP4, SUSD2, TFRC, TGFBI, TGM2, TIMP1, TKT, TM7SF3, TOLLIP, TPST2, TUBB, UBA1, VASN, VASP, VDAC1, VDAC2, WARS1, WDR1, YWHAB*** | 1.59E-10**<br>2.78E-03 |

KEGG, Kyoto Encyclopaedia of Genes and Genomes; and DEGs, differentially expressed genes. Gene symbols not available replaced with ensembl ID. DEGs in analysis: 1310, up-regulated genes (n = 795), down-regulated genes (n = 515), exclusively up-regulated genes (n = 115), and exclusively down-regulated genes (n = 40). Up-regulated DEGs in bold, down-regulated DEGs in non-bold, exclusive DEGs are underlined. *FDR for total DEGs (up- and down-regulated together), **FDR for up-regulated DEGs, and ***FDR for down-regulated DEGs. FDR for exclusive DEGs (up- or downregulated) are underlined. Data based on average of logarithmic scale base two ($log_2$). Data analysed with DAVID (https://david.ncifcrf.gov/).

**Supplementary table 9.** Differentially expressed genes in Luminal B *f*HER2 negative-derived cell lines associated with selected KEGG pathways.

| Category | Term | Genes | FDR |
|---|---|---|---|
| Metabolic-related | Metabolic pathways (fca01100) | AASS, ABAT, ACACA, ACACB, ACADL, ACER2, **ACLY**, ACO1, **ACO2**, ACOX2, ACSBG1, **ACSL3**, **ACSL6**, ACSM1, ACSM4, ADA, ADA2, ADH4, ADK, **ADO**, **ADSL**, AGK, AGL, AGMAT, **AGPAT2**, AGPS, **AHCY**, **AK2**, AK5, **AK6**, AK8, **AKR1A1**, AKR1C3, **AKR1D1**, ALAS2, **ALDH18A1**, **ALDH1A3**, **ALDH3B1**, **ALDH5A1**, **ALG1**, **ALG12**, ALG14, **ALG2**, **ALG3**, ALG9, **ALOX15B**, ALOX5, ALPL, **AMD1**, **AMPD2**, AMY2B, AOX1, ARG1, ASAH1, ASAH2, **ASNS**, **ASPA**, **ASS1**, **ATIC**, **ATP5F1A**, **ATP5F1B**, **ATP5F1C**, **ATP5MC3**, **ATP5PB**, **ATP5PD**, **ATP5PF**, **ATP5PO**, **ATP6V0B**, ATP6V1B1, **ATP6V1E1**, AZIN2, B3GALT5, **B3GNT4**, B3GNT5, **B4GALT2**, B4GALT6, **B4GAT1**, **BAAT**, **BCAT1**, BST1, **C1GALT1C1**, **CAD**, **CBS**, CDS1, **CERS2**, CERS3, **CHKB**, CKM, CMBL, CNDP1, **COMT**, COQ3, **COQ6**, **COQ7**, **COX17**, COX4I2, **COX5B**, **CS**, CSGALNACT1, **CYC1**, **CYP19A1**, CYP21A2, **CYP27A1**, **CYP27B1**, **CYP2J2**, CYP2R1, **CYP51A1**, CYP7A1, **DAD1**, **DCK**, **DCT**, **DDOST**, **DEGS1**, DGKG, DGKH, DGKI, **DGKQ**, **DGKZ**, **DHCR24**, **DHCR7**, **DHFR**, **DHRS3**, **DLAT**, **DLST**, DMGDH, **DNMT1**, DNMT3A, **DOLK**, **DPAGT1**, **DPM2**, DPYS, DSE, **DUT**, **EBP**, **ENO1**, ENPP1, ENPP3, **ENSFCAG00000002620**, ENSFCAG00000011675, **ENSFCAG00000023226**, ENSFCAG00000023835, ENSFCAG00000024210, **ENSFCAG00000025768**, ENSFCAG00000027562, ENSFCAG00000028790, **ENSFCAG00000029444**, ENSFCAG00000029906, ENSFCAG00000029956, ENSFCAG00000030205, EPHX2, **EPRS1**, **FAH**, **FAHD1**, **FASN**, FAXDC2, FBP1, FBP2, **FH**, **FLAD1**, FOLH1, **G6PC3**, GAD1, GAD2, GADL1, GALC, **GALE**, **GALK1**, GALM, GALNT10, GALNT11, GALNT12, GALNT14, GALNT15, GALNT17, GALNT3, GALNT5, GALNTL5, GANC, **GAPDH**, **GART**, GATB, GATM, GBE1, **GCNT1**, GCNT2, GDA, GFPT2, GGT1, GGT5, GK, GLDC, **GLS2**, GMDS, **GNPDA1**, **GOT1**, **GOT2**, GPAT3, **GPAT4**, **GRHPR**, GSS, **GSTZ1**, **HADH**, **HADHA**, HAL, **HCN3**, HDC, HGD, HIBADH, HIBCH, **HK1**, **HK2**, HLCS, **HMBS**, **HMGCR**, **HMGCS1**, HOGA1, HPD, **HPRT1**, HSD11B1, **HSD17B1**, HSD3B2, **HYAL2**, **HYAL3**, **IDH1**, **IDH3A**, **IDH3B**, **IDI1**, **IMPDH2**, INPP4A, INPP5B, **INPP5E**, **INPP5J**, ITPKB, KYAT3, KYNU, **LAP3**, LCT, **LDHA**, LIPC, **LPCAT4**, **LPIN1**, **LTA4H**, LTC4S, MAN1A2, MAN1C1, MAN2A1, **MAN2A2**, MAOA, MAOB, **MAT1A**, MBOAT2, **MCAT**, MCCC1, **MDH1**, **MDH2**, **ME1**, ME3, MGAM, **MGAT1**, **MGAT2**, MGAT4A, **MGAT4B**, MGAT4C, MGAT5, MGAT5B, MGLL, MOCS1, **MOGS**, **MTHFD1**, **MTHFD2**, MTM1, MTMR7, MTR, **MVD**, NADK2, **NAGS**, NAMPT, NDST3, NDST4, **NDUFA11**, **NDUFA12**, **NDUFA2**, **NDUFA8**, **NDUFAB1**, **NDUFB10**, **NDUFB11**, **NDUFB6**, **NDUFS1**, **NDUFS2**, **NDUFS5**, **NDUFS8**, **NDUFV1**, **NME6**, **NMNAT1**, NOS2, NOS3, NT5C2, NT5C3A, **NT5C3B**, **OCRL**, **ODC1**, **P4HA2**, P4HA3, **PAFAH1B3**, **PAPSS1**, **PAPSS2**, PCCA, PCK1, **PCK2**, | 6.60E-05*<br>6.40E-09**<br>1.43E-03**<br>5.56E-03**** |

| | | | |
|---|---|---|---|
| | | **PDHA1**, PDXK, **PFAS**, **PFKL**, **PFKP**, PGAM2, PGAP1, **PGD**, **PGK1**, **PHGDH**, **PIGB**, **PIGC**, PIGK, PIGL, **PIGM**, PIGN, **PIGQ**, **PIGT**, **PIGW**, **PIGY**, PIK3C2G, PIK3C3, PIP5K1B, **PISD**, **PKM**, **PLA2G3**, PLA2G4D, PLA2G4E, PLA2G5, PLCB1, PLCB2, **PLCB3**, PLCB4, **PLCD1**, **PLCD3**, **PLCG2**, PLCZ1, PLD4, PLPP1, PLPP3, PMM2, PNLIPRP3, PNMT, POLA1, **POLA2**, **POLD1**, **POLE**, **POLE3**, **POLR1C**, **POLR1H**, **POLR2B**, **POLR2E**, **POLR2F**, **POLR2G**, **POLR2H**, **POLR2J**, **POLR3C**, PRIM2, **PRPS1**, **PRPS2**, **PSAT1**, **PTGES**, **PTGES2**, PTGIS, PTGS1, **PYCR2**, PYGM, **QARS1**, RDH12, RDH14, RGN, **RPN1**, **RPN2**, **RRM1**, **RRM2**, **RRM2B**, **SAT1**, **SDHA**, **SDHD**, **SEPHS1**, SGMS1, SGMS2, SGPL1, **SHMT1**, **SHMT2**, **SMPD1**, SMPD3, **SPTLC1**, **SQLE**, **SRM**, **ST3GAL4**, ST6GAL1, ST6GAL2, ST6GALNAC5, **STT3A**, **STT3B**, SUCLG2, SYNJ1, **TALDO1**, TDO2, **TK1**, **TKT**, TPH1, **TPI1**, TPK1, **TST**, **TYMS**, TYR, UAP1, **UCK2**, UGCG, UGP2, **UMPS**, **UQCRB**, **UQCRC1**, **UQCRFS1**, **UROD**, XYLB | |
| | Purine metabolism (fca00230) | ADA, ADA2, ADCY10, ADCY2, **ADCY3**, ADCY4, ADCY5, ADCY8, ADCY9, ADK, **ADSL**, **AK2**, AK5, **AK6**, AK8, **AMPD2**, **ATIC**, **DCK**, ENPP1, ENPP3, ENSFCAG00000024210, ENSFCAG00000029956, ENTPD1, ENTPD3, ENTPD6, **GART**, GDA, **GMPR2**, GUCY1A1, GUCY1B1, GUCY2C, GUCY2D, GUCY2F, **HCN3**, **HPRT1**, **IMPDH2**, **NME6**, NT5C2, NT5C3A, **NT5C3B**, **PAPSS1**, **PAPSS2**, PDE10A, PDE11A , PDE1A, PDE2A, PDE4B, PDE4D, PDE7A, PDE7B, PDE8B, **PFAS**, **PKM**, POLA1, **POLA2**, **POLD1**, **POLE**, **POLE3**, **POLR1C**, **POLR1H**, **POLR2B**, **POLR2E**, **POLR2F**, **POLR2G**, **POLR2H**, **POLR2J**, **POLR3C**, PRIM2, **PRPS1**, **PRPS2** , RDH14, **RRM1**, **RRM2**, **RRM2B** | 1.08E-02* |
| | Tyrosine metabolism (fca00350) | ADH4, **ALDH1A3**, **ALDH3B1**, AOX1, **COMT**, **DCT**, ENSFCAG00000030205, **FAH**, **FAHD1**, **GOT1**, **GOT2**, **GSTZ1**, HGD, HPD, MAOA, MAOB, **MIF**, PNMT, TYR | 2.01E-02* |
| | N-Glycan biosynthesis (fca00510) | **ALG1**, **ALG12**, ALG14, **ALG2**, **ALG3**, ALG9, **B4GALT2**, **DAD1**, **DDOST**, **DOLK**, **DPAGT1**, **DPM2**, MAN1C1, MAN1A2, MAN2A1, **MAN2A2**, **MGAT1**, **MGAT2**, MGAT4A, **MGAT4B**, MGAT4C, MGAT5, MGAT5B, **MOGS**, **RPN1**, **RPN2**, ST6GAL1, ST6GAL2, **STT3A**, **STT3B** | 8.88E-04* |
| | Biosynthesis of amino acids (fca01230) | ACO1, **ACO2**, **ALDH18A1**, ARG1, **ASS1**, **BCAT1**, **CBS**, **CS**, **ENO1**, **ENSFCAG00000023226**, **GAPDH**, **GOT1**, **GOT2**, **HCN3**, **IDH1**, **IDH3A**, **IDH3B**, **MAT1A**, MTR, **NAGS**, **PFKL**, **PFKP**, PGAM2, **PGK1**, **PHGDH**, **PKM**, **PRPS1**, **PRPS2**, **PSAT1**, **PYCR2**, **SHMT1**, **SHMT2**, **TALDO1**, **TKT**, **TPI1** | 1.08E-02*<br>8.74E-08**<br>3.85E-03** |
| | Citrate cycle/TCA cycle (fca00020) | **ACLY**, ACO1, **ACO2**, **CS**, **DLAT**, **DLST**, **FH**, **IDH1**, **IDH3A**, **IDH3B**, **MDH1**, **MDH2**, PCK1, **PCK2**, **PDHA1**, **SDHA**, **SDHD**, SUCLG2 | 1.61E-02*<br>1.86E-04**<br>1.03E-03** |
| | Carbon metabolism (fca01200) | ACO1, **ACO2**, **CS**, **DLAT**, **DLST**, **ENO1**, FBP1, FBP2, **FH**, **GAPDH**, GLDC, **GOT1**, **GOT2**, **HADHA**, **HCN3**, HIBCH, **HK1**, **HK2**, **IDH1**, **IDH3A**, **IDH3B**, **MDH1**, **MDH2**, **ME1**, ME2, ME3, PCCA, **PDHA1**, **PFKL**, **PFKP**, PGAM2, **PGD**, **PGK1**, **PHGDH**, **PKM**, **PRPS1**, **PRPS2**, **PSAT1**, RGN, **SDHA**, **SDHD**, **SHMT1**, **SHMT2**, SUCLG2, **TALDO1**, **TKT**, **TPI1** | 3.95E-02*<br>1.80E-05** |
| | Oxidative phosphorylation | **ATP5F1A**, **ATP5F1B**, **ATP5F1C**, **ATP5MC3**, **ATP5PB**, **ATP5PD**, **ATP5PF**, **ATP5PO**, **ATP6V0B**, **ATP6V1E1**, | 1.22E-03**<br>4.13E-06** |

| | | | |
|---|---|---|---|
| | (fca00190) | ***COX17***, ***COX5B***, ***COX7A2***, ***CYC1***, ***ENSFCAG00000002620***, ***ENSFCAG00000029444***, ***NDUFA11***, ***NDUFA12***, ***NDUFA2***, ***NDUFA8***, ***NDUFAB1***, ***NDUFB10***, ***NDUFB11***, ***NDUFB6***, ***NDUFS1***, ***NDUFS2***, ***NDUFS5***, ***NDUFS8***, ***NDUFV1***, ***PPA1***, ***SDHA***, ***SDHD***, ***UQCRB***, ***UQCRC1***, ***UQCRFS1*** | |
| | 2-Oxocarboxylic acid metabolism (fca01210) | ***ACO2***, ***BCAT1***, ***CS***, ***ENSFCAG00000023226***, ***GOT1***, ***GOT2***, ***IDH1***, ***IDH3A***, ***IDH3B***, ***NAGS*** | 2.26E-03**<br>2.04E-02** |
| | Pyrimidine metabolism (fca00240) | ***CAD***, ***DCK***, ***DUT***, ***NME6***, ***NT5C3B***, ***POLA2***, ***POLD1***, ***POLE***, ***POLE3***, ***POLR1C***, ***POLR1H***, ***POLR2B***, ***POLR2E***, ***POLR2F***, ***POLR2G***, ***POLR2H***, ***POLR2J***, ***POLR3C***, ***RRM1***, ***RRM2***, ***RRM2B***, ***TK1***, ***TYMS***, ***UCK2***, ***UMPS*** | 1.08E-02** |
| | One carbon pool by folate (fca00670) | ***ALDH1L2***, ***ATIC***, ***DHFR***, ***GART***, ***MTHFD1***, ***MTHFD2***, ***SHMT1***, ***SHMT2***, ***TYMS*** | 1.89E-02** |
| | Glycolysis / Gluconeogenesi s (fca00010) | ***AKR1A1***, ***ALDH1A3***, ***ALDH3B1***, ***DLAT***, ***ENO1***, ***G6PC3***, ***GAPDH***, ***HCN3***, ***HK1***, ***HK2***, ***LDHA***, ***PCK2***, ***PDHA1***, ***PFKL***, ***PFKP***, ***PGK1***, ***PKM***, ***TPI1*** | 2.40E-02** |
| | Pyruvate metabolism (fca00620) | ***ACYP1***, ***DLAT***, ***FH***, ***GLO1***, ***GRHPR***, ***HCN3***, ***LDHA***, ***MDH1***, ***MDH2***, ***ME1***, ***PCK2***, ***PDHA1***, ***PKM*** | 4.23E-02** |
| | Starch and sucrose metabolism (fca00500) | *AGL*, *AMY2B* , *ENPP1*, *ENPP3*, *GANC* , *GBE1*, *GYS2*, *MGAM*, *PGM2L1*, *UGP2* | 2.10E-02*** |
| Cell growth and death | Cell cycle (fca04110) | *ABL1*, *ANAPC10*, ***ANAPC2***, *ATM*, *ATR*, ***BUB1***, ***BUB1B***, ***BUB3***, ***CCNA2***, ***CCNB1***, ***CCNB2***, *CDC14A*, *CDC14B*, ***CDC25A***, ***CDC25B***, ***CDC45***, ***CDC6***, ***CDK1***, ***CDK2***, ***CDK4***, ***CDKN1A***, ***CDKN2A***, ***CDKN2D***, ***CHEK1***, *CUL1*, ***E2F2***, ***E2F4***, ***ELOVL1***, ***ESPL1***, ***FZR1***, ***HDAC1***, ***HDAC2***, *MAD1L1*, ***MAD2L1***, ***MCM2***, ***MCM3***, ***MCM4***, ***MCM5***, ***MCM6***, ***MCM7***, ***MDM2***, ***ORC1***, *ORC4*, *ORC5*, ***ORC6***, ***PCNA***, ***PKMYT1***, ***PLK1***, *PRKDC*, ***PTTG1***, ***RAD21***, *RB1*, *RBL1*, *SMAD2*, ***SMAD3***, *STAG1*, *STAG2*, ***TFDP1***, *TFDP2*, ***TP53***, ***TTK***, ***YWHAB***, ***YWHAE***, ***YWHAG***, ***ZBTB17*** | 6.60E-05*<br>2.61E-09** |
| | p53 signalling pathway (fca04115) | *ATM*, *ATR*, ***BAX***, ***BID***, *CASP8*, ***CCNB1***, ***CCNB2***, *CD82*, ***CDK1***, ***CDK2***, ***CDK4***, ***CDKN1A***, ***CDKN2A***, ***CHEK1***, *COP1*, ***EI24***, ***GTSE1***, *IGF1*, ***MDM2***, *MDM4*, ***PERP***, *PTEN*, *RCHY1*, ***RRM2***, ***RRM2B***, ***SERPINB5***, *SESN1*, ***SESN2***, *SESN3*, ***STEAP3***, ***THBS1***, ***TP53***, ***TP53I3*** | 1.71E-02*<br>2.26E-03** |
| Transcription | Spliceosome (fca03040) | ***BUD31***, *CCDC12*, ***CDC5L***, ***CHERP***, ***DDX23***, ***DDX39B***, ***DHX16***, ***DHX38***, ***EFTUD2***, ***EIF4A3***, ***HNRNPA1***, ***HNRNPC***, ***HNRNPM***, ***HSPA2***, ***HSPA8***, ***LSM7***, ***MAGOH***, ***PCBP1***, ***PHF5A***, ***PLRG1***, ***PQBP1***, *PRPF18*, ***PRPF19***, ***PRPF31***, ***PRPF4***, ***PRPF6***, ***PRPF8***, ***RBM17***, ***RBM22***, ***RBMX***, ***SART1***, ***SF3A1***, ***SF3A3***, ***SF3B2***, ***SF3B4***, ***SF3B5***, ***SF3B6***, ***SNRNP200***, ***SNRPA***, ***SNRPB2***, ***SNRPD1***, ***SNRPD2***, ***SNU13***, ***SNW1***, *THOC2*, *TRA2A*, ***TXNL4A***, ***U2AF1***, ***USP39***, ***WBP11***, ***XAB2***, ***ZMAT2*** | 4.96E-02*<br>5.56E-03****<br>2.61E-09**<br>1.01E-11** |
| Translation | mRNA surveillance pathway (fca03015) | ***CLP1***, ***CPSF2***, ***CSTF1***, ***DDX39B***, ***ETF1***, ***MAGOH***, ***NUDT21***, ***NXT1***, ***PABPC4***, ***PPP2R1A***, ***PPP2R5D***, ***PYM1***, ***SYMPK***, ***UPF1***, ***UPF3A***, ***UPF3B*** | 3.51E-02** |

| | | | |
|---|---|---|---|
| | RNA transport (fca03013) | ***AAAS, DDX20, DDX39B, EEF1A1, EIF2B1, EIF2B2, EIF2B5, EIF2S1, EIF2S3, EIF3A, EIF3B, EIF3D, EIF3F, EIF3G, EIF3I, EIF4A3, EIF4B, EIF4EBP1, EIF4G1, EIF5, FXR2, GEMIN4, GEMIN5, GEMIN6, KPNB1, MAGOH, NUP107, NUP43, NUP50, NXT1, PABPC4, PHAX, POP7, PRMT5, PYM1, RAN, RANGAP1, RNPS1, RPP25, RPP25L, RPP38, STRAP, TACC3, THOC5, THOC7, UBE2I, UPF1, UPF3A, UPF3B, XPOT*** | 6.15E-07**<br>2.06E-06** |
| | Ribosome biogenesis in eukaryotes (fca03008) | ***AK6, BMS1, CSNK2B, EIF6, EMG1, FBL, GNL2, GNL3, GTPBP4, IMP4, MPHOSPH10, NHP2, NOB1, NOL6, NOP56, NOP58, NXT1, POP7, PWP2, RAN, RPP25, RPP25L, RPP38, SNU13, TBL3, UTP14A, UTP15, WDR3*** | 1.49E-05**<br>5.82E-05** |
| | Aminoacyl-tRNA biosynthesis (fca00970) | ***AARS1, EPRS1, GARS1, HARS1, HARS2, IARS1, KARS1, LARS1, MARS1, MARS2, NARS1, PARS2, QARS1, RARS1, SARS1, TARS1, TARS2, VARS1, WARS1, YARS1*** | 5.07E-05** |
| | Ribosome (fca03010) | ***DNAJC9, ENSFCAG00000022178, MRPL10, MRPL11, MRPL16, MRPL18, MRPL19, MRPS12, MRPS15, RPL12, RPL18, RPL24, RPL26, RPL27A, RPL29, RPL3, RPL30, RPL35, RPL36AL, RPL4, RPL8, RPLP0, RPS10, RPS15A, RPS20, RPS26, RPS3, RPS5, RPS6, RPS7, RPS9, RPSA*** | 2.77E-02**<br>1.86E-03** |
| Replication and repair | DNA replication (fca03030) | ***FEN1, MCM2, MCM3, MCM4, MCM5, MCM6, MCM7, PCNA, POLA2, POLD1, POLE, POLE3, RFC2, RFC3, RNASEH2A, RPA1*** | 4.77E-04** |
| | Base excision repair (fca03410) | ***APEX1, FEN1, LIG3, MPG, PARP1, PARP3, PCNA, POLD1, POLE, POLE3, SMUG1, TDG, XRCC1*** | 1.73E-02** |
| Protein processing | Protein processing in endoplasmic reticulum (fca04141) | ***ATF4***, ***BAG1***, ***BAX***, ***BMP6***, ***CALR***, ***CANX***, ***CAPN1***, *CASP12*, *CUL1*, ***DAD1***, ***DDIT3***, ***DDOST***, ***DERL1***, ***DERL2***, ***DNAJA2***, ***DNAJB11***, ***DNAJC3***, *DNAJC5B*, *EDEM2*, *EDEM3*, *EIF2AK4*, ***EIF2S1***, *ERN1*, *ERO1B*, ***ERP29***, ***HERPUD1***, ***HSP90AA1***, ***HSP90AB1***, ***HSP90B1***, ***HSPA2***, ***HSPA5***, ***HSPA8***, ***HSPBP1***, ***HSPH1***, ***HYOU1***, ***LMAN2***, *MAN1A2*, *MAN1C1*, *MAP3K5*, *MAPK10*, *MAPK8*, *MARCHF6*, ***MOGS***, *NGLY1*, ***OS9***, ***P4HB***, ***PDIA3***, ***PDIA4***, ***PDIA6***, ***PREB***, ***PRKCSH***, ***RPN1***, ***RPN2***, *SEC24B*, *SEC24D*, *SEC31A*, *SEC31B*, ***SEC61A1***, ***SEL1L***, *SEL1L2*, ***SELENOS***, ***SSR1***, ***SSR3***, ***SSR4***, ***STT3A***, ***STT3B***, ***TRAF2***, ***TRAM1***, *UBE2E2*, ***UBE2J1***, *UBE4B*, ***VCP*** | 6.68E-03*<br>1.33E-06** |
| | Proteasome (fca03050) | ***POMP, PSMA1, PSMA6, PSMA7, PSMB1, PSMB3, PSMB4, PSMB5, PSMC1, PSMC2, PSMC4, PSMC6, PSMD13, PSMD2, PSMD3, PSMD6, PSMD8, PSME2, PSME3*** | 1.86E-04**<br>7.02E-03** |
| Adhesion, and cell interaction | ECM-receptor interaction (fca04512) | *CD36*, *CD47*, ***COL11A2***, *COL1A1*, *COL1A2*, ***COL2A1***, *COL3A1*, ***COL4A1***, *COL4A4*, *COL4A6*, *COL6A1*, *COL6A3*, *COL6A6*, *ENSFCAG00000029640*, ***HMMR***, ***HSPG2***, *ITGA1*, *ITGA11*, *ITGA2B*, ***ITGA3***, *ITGA4*, *ITGA5*, *ITGA6*, *ITGA8*, *ITGA9*, ***ITGB1***, *ITGB3*, ***ITGB4***, *ITGB6*, *ITGB8*, ***FN1***, *LAMA2*, *LAMA3*, ***LAMA5***, ***LAMB1***, *LAMB4*, *LAMC2*, *LAMC3*, *SV2A*, *SV2B*, ***THBS1***, *THBS2*, ***THBS3***, *TNC*, *VTN*, *VWF* | 2.01E-03*<br>8.35E-05***<br>4.77E-02*** |
| | Focal adhesion (fca04510) | ***ACTN1***, *ACTN2*, *ACTN3*, *AKT3*, ***ARHGAP35***, ***BCAR1***, *BRAF*, ***COL11A2***, *COL1A1*, *COL1A2*, ***COL2A1***, *COL3A1*, ***COL4A1***, *COL4A4*, *COL4A6*, *COL6A1*, *COL6A3*, *COL6A6*, ***CTNNB1***, *DIAPH1*, *DOCK1*, *EGF*, *EGFR*, ***FLNA***, *FLNC*, ***FN1***, *HGF*, ***HRAS***, *IGF1*, ***ILK***, *ITGA1*, *ITGA11*, *ITGA2B*, ***ITGA3***, *ITGA4*, *ITGA5*, *ITGA6*, *ITGA8*, *ITGA9*, ***ITGB1***, *ITGB3*, ***ITGB4***, *ITGB6*, *ITGB8*, *KDR*, *LAMA2*, *LAMA3*, ***LAMA5***, ***LAMB1***, *LAMB4*, *LAMC2*, *LAMC3*, *MAPK10*, | 2.01E-03*<br>7.65E-06***<br>4.46E-02*** |

| | | | |
|---|---|---|---|
| | | *MAPK8*, ***MYL12A***, ***MYLK3***, ***PAK4***, *PAK5*, *PAK6*, *PARVB*, *PARVG*, ***PDGFB***, *PDGFC*, *PDGFD*, *PDGFRA*, *PDGFRB*, *PIK3CA*, *PIK3CG*, *PIK3R1*, *PRKCA*, *PRKCG*, *PTEN*, *PTK2*, *RAC2*, ***RAP1B***, *ROCK1*, *SOS1*, *SOS2*, *SRC*, ***THBS1***, *THBS2*, ***THBS3***, ***TLN1***, *TNC*, ***VASP***, *VAV3*, *VCL*, ***VEGFA***, *VEGFC*, *VTN*, *VWF* | |
| | Gap junction (fca04540) | *ADCY2*, ***ADCY3***, *ADCY4*, *ADCY5*, *ADCY8*, *ADCY9*, ***CDK1***, *EGF*, *EGFR*, ***GJA1***, *GNA14*, *GUCY1A1*, *GUCY1B1*, ***HRAS***, *HTR2B*, *ITPR1*, *ITPR2*, ***ITPR3***, *KRAS*, *LPAR1*, *MAPK7*, ***PDGFB***, *PDGFC*, *PDGFD*, *PDGFRA*, *PDGFRB*, *PLCB1*, *PLCB2*, ***PLCB3***, *PLCB4*, *PRKCA*, *PRKCG*, *SOS1*, *SOS2*, *SRC*, ***TUBAL3***, ***TUBB***, ***TUBB4A*** | 3.65E-02* 1.66E-03*** |
| | Cell adhesion molecules (CAMs) (fca04514) | *CD2*, *CD226*, *CD28*, *CD34*, *CD40*, *CD80*, *CD86*, *CDH4*, *CDH5*, *CLDN4*, *CLDN7*, *CLDN8*, *CNTN1*, *CNTN2*, *CNTNAP1*, *CTLA4*, *ENSFCAG00000009202*, *ENSFCAG00000009442*, *ITGA4*, *ITGA6*, *ITGA8*, *ITGA9*, *ITGAM*, *ITGB2*, *ITGB8*, *JAM2*, *LRRC4*, *NCAM2*, *NFASC*, *NLGN4X*, *OCLN*, *PDCD1LG2*, *PECAM1*, *PTPRC*, *PTPRM*, *SELL*, *SIGLEC1*, *VCAM1*, *VCAN*, *VTCN1* | 1.74E-03*** |
| Cell movement | Regulation of actin cytoskeleton (fca04810) | *ABI2*, ***ACTN1***, *ACTN2*, *ACTN3*, *APC*, ***ARHGAP35***, *ARHGEF12*, *ARHGEF4*, *ARHGEF6*, *ARHGEF7*, ***ARPC1A***, ***ARPC4***, ***ARPC5L***, ***BCAR1***, *BRAF*, ***BRK1***, ***CFL1***, ***CFL2***, *DIAPH1*, *DOCK1*, *EGF*, *EGFR*, ***EZR***, *FGD3*, *FGF12*, *FGF13*, ***FGF21***, *FGF7*, *FGFR1*, ***FN1***, ***GIT1***, ***GNA13***, ***HRAS***, *IQGAP2*, ***IQGAP3***, *ITGA1*, *ITGA11*, *ITGA2B*, ***ITGA3***, *ITGA4*, *ITGA5*, *ITGA6*, *ITGA8*, *ITGA9*, ***ITGAE***, *ITGAM*, ***ITGB1***, *ITGB2*, *ITGB3*, ***ITGB4***, *ITGB6*, *ITGB8*, *KRAS*, *LIMK2*, ***MRAS***, ***MSN***, ***MYL12A***, ***MYLK3***, *NCKAP1L*, ***PAK4***, *PAK5*, *PAK6*, ***PDGFB***, *PDGFC*, *PDGFD*, *PDGFRA*, *PDGFRB*, *PIK3CA*, *PIK3CG*, *PIK3R1*, *PIP4K2A*, ***PIP4K2C***, *PIP5K1B*, *PTK2*, *RAC2*, ***RDX***, *ROCK1*, *SCIN*, *SOS1*, *SOS2*, *SRC*, *SSH2*, *TIAM1*, *VAV3*, *VCL*, *WAS*, *WASF2* | 8.77E-03* 2.71E-04*** 1.21E-02*** |
| Signal transduction | PI3K-Akt signalling pathway (fca04151) | *AKT3*, *ANGPT4*, *ATF2*, ***ATF4***, ***BRCA1***, ***CDC37***, ***CDK2***, ***CDK4***, ***CDKN1A***, ***COL11A2***, *COL1A1*, *COL1A2*, ***COL2A1***, *COL3A1*, ***COL4A1***, *COL4A4*, *COL4A6*, *COL6A1*, *COL6A3*, *COL6A6*, *CREB1*, *CREB3L1*, *CREB3L2*, *CREB5*, ***CRTC2***, *CSF1R*, ***DDIT4***, *EGF*, *EGFR*, ***EIF4B***, ***EIF4EBP1***, ***EPHA2***, *FGF12*, *FGF13*, ***FGF21***, *FGF7*, *FGFR1*, ***FN1***, ***G6PC3***, *GHR*, ***GYS1***, *GYS2*, *HGF*, ***HRAS***, ***HSP90AA1***, ***HSP90AB1***, ***HSP90B1***, *IGF1*, *IL2RA*, *IL2RB*, *IL4*, *IL4R*, *IL6R*, *IL7R*, *INSR*, *ITGA1*, *ITGA11*, *ITGA2B*, ***ITGA3***, *ITGA4*, *ITGA5*, *ITGA6*, *ITGA8*, *ITGA9*, ***ITGB1***, *ITGB3*, ***ITGB4***, *ITGB6*, *ITGB8*, *JAK1*, *JAK2*, *JAK3*, *KDR*, *KIT*, *KRAS*, *LAMA2*, *LAMA3*, ***LAMA5***, ***LAMB1***, *LAMB4*, *LAMC2*, *LAMC3*, *LPAR1*, *LPAR6*, ***MDM2***, *NFKB1*, *NOS3*, *OSMR*, *PCK1*, ***PCK2***, ***PDGFB***, *PDGFC*, *PDGFD*, *PDGFRA*, *PDGFRB*, *PIK3AP1*, *PIK3CA*, *PIK3CG*, *PIK3R1*, ***PKN1***, ***PPP2R1A***, *PPP2R2A*, *PPP2R2B*, *PPP2R3A*, *PPP2R5A*, ***PPP2R5D***, *PRKAA2*, *PRL*, *PRLR*, *PTEN*, *PTK2*, *RHEB*, ***RPS6***, *RPTOR*, *SOS1*, *SOS2*, ***STK11***, *SYK*, *TEK*, ***THBS1***, *THBS2*, ***THBS3***, *TLR4*, *TNC*, ***TP53***, ***VEGFA***, *VEGFC*, *VTN*, *VWF*, ***YWHAB***, ***YWHAE***, ***YWHAG*** | 1.57E-02* 9.74E-05*** |
| | MAPK signalling pathway (fca04010) | *AKT3*, *ARRB1*, *ATF2*, ***ATF4***, *BRAF*, *CACNA1A*, *CACNA1C*, *CACNA1D*, *CACNA2D1*, *CACNA2D2*, *CACNA2D3*, ***CACNA2D4***, ***CACNB3***, *CACNB4*, *CACNG2*, *CACNG7*, *CD14*, ***CDC25B***, ***DDIT3***, *DUSP16*, ***DUSP4***, ***DUSP7***, ***ECSIT***, *EGF*, *EGFR*, *FGF12*, *FGF13*, ***FGF21***, *FGF7*, *FGFR1*, ***FLNA***, | 2.13E-02* 4.95E-05*** 1.21E-02*** |

| | | | |
|---|---|---|---|
| | | FLNC, **HRAS**, **HSPA2**, **HSPA8**, IL1B, KRAS, MAP2K6, MAP3K1, **MAP3K12**, MAP3K14, MAP3K20, MAP3K3, MAP3K5, MAP3K8, MAP4K3, MAPK10, **MAPK11**, MAPK14, MAPK7, MAPK8, **MAPKAPK2**, **MAPKAPK3**, MECOM, MEF2C, **MKNK2**, **MRAS**, NF1, NFATC3, NFKB1, NLK, NTF3, NTF4, **NTRK1**, NTRK2, **PDGFB**, PDGFRA, PDGFRB, PLA2G4D, PLA2G4E, PPM1B, PPP3CA, PPP3CB, PPP3CC, **PPP5C**, PRKCA, PRKCG, PTPN5, RAC2, **RAP1B**, RASA1, RASA2, RASGRP3, RASGRP4, RPS6KA3, RPS6KA5, RPS6KA6, SOS1, SOS2, **TAB1**, TAOK1, TAOK3, TGFBR2, TNF, **TP53**, **TRAF2** | |
| | Rap1 signalling pathway (fca04015) | ADCY2, **ADCY3**, ADCY4, ADCY5, ADCY8, ADCY9, ADORA2A, AKT3, ANGPT4, APBB1IP, ARAP3, **BCAR1**, BRAF, CSF1R, **CTNNB1**, DOCK4, EGF, EGFR, ENSFCAG00000001479, **EPHA2**, FGF12, FGF13, **FGF21**, FGF7, FGFR1, FYB1, GNA14, GRIN2A, GRIN2B, HGF, **HRAS**, **ID1**, IGF1, INSR, ITGA2B, ITGAM, **ITGB1**, ITGB2, ITGB3, KDR, KIT, KRAS, KRIT1, LCP2, LPAR1, MAGI3, MAP2K6, **MAPK11**, MAPK14, **MRAS**, **P2RY1**, PARD3, **PDGFB**, PDGFC, PDGFD, PDGFRA, PDGFRB, PIK3CA, PIK3CG, PIK3R1, PLCB1, PLCB2, **PLCB3**, PLCB4, PRKCA, PRKCG, PRKD1, RAC2, **RAP1B**, RAPGEF3, RAPGEF4, RASGRP3, RASSF5, RGS14, SIPA1L2, SKAP1, SRC, TEK, **THBS1**, TIAM1, **TLN1**, **VASP**, **VEGFA**, VEGFC | 2.06E-02*<br>7.65E-06***<br>9.67E-03*** |
| | cGMP-PKG signalling pathway (fca04022) | ADCY2, **ADCY3**, ADCY4, ADCY5, ADCY8, ADCY9, ADRA1A, AGTR1, AKT3, ATF2, **ATF4**, **ATP1A1**, ATP1A4, **ATP1B3**, ATP2A3, ATP2B2, **ATP2B4**, CACNA1C, CACNA1D, CNGA1, CREB1, CREB3L1, CREB3L2, CREB5, EDNRB, **ENSFCAG00000001211**, ENSFCAG00000002691, **GNA13**, GNA14, GTF2I, GUCY1A1, GUCY1B1, INSR, IRAG1, ITPR1, ITPR2, **ITPR3**, KCNJ8, KCNMB4, MEF2A, MEF2C, **MYLK3**, NFATC3, NOS3, PDE2A, PLCB1, PLCB2, **PLCB3**, PLCB4, **PLN**, **PPIF**, PPP3CA, PPP3CB, PPP3CC, PRKCE, RGS2, ROCK1, **SLC25A5**, SLC8A1, TRPC6, **VASP**, **VDAC1**, **VDAC2**, **VDAC3** | 4.73E-02*<br>4.82E-04*** |
| | Sphingolipid signalling pathway (fca04071) | ACER2, AKT3, ASAH1, ASAH2, **BAX**, **BID**, **CERS2**, CERS3, **DEGS1**, FCER1A, GAB2, **GNA13**, GNA14, **HRAS**, KRAS, MAP3K5, MAPK10, **MAPK11**, MAPK14, MAPK8, NFKB1, NOS3, PIK3CA, PIK3CG, PIK3R1, PLCB1, PLCB2, **PLCB3**, PLCB4, **PPP2R1A**, PPP2R2A, PPP2R2B, PPP2R3A, PPP2R5A, **PPP2R5D**, PRKCA, PRKCE, PRKCG, PTEN, RAC2, ROCK1, S1PR1, **S1PR2**, S1PR3, SGMS1, SGMS2, SGPL1, **SMPD1**, **SPTLC1**, TNF, **TP53**, **TRAF2** | 3.55E-02*<br>1.52E-03***<br>1.21E-02*** |
| | Calcium signalling pathway (fca04020) | ADCY2, **ADCY3**, ADCY4, ADCY8, ADCY9, ADORA2A, ADRA1A, AGTR1, ATP2A3, ATP2B2, **ATP2B4**, AVPR1A, CACNA1A, CACNA1C, CACNA1D, CAMK2A, CAMK2B, CAMK2D, CAMK2G, CAMK4, CYSLTR1, EDNRB, EGFR, **ENSFCAG00000001211**, **ERBB3**, GNA14, GNA15, GRIN2A, GRPR, HTR2B, ITPKB, ITPR1, ITPR2, **ITPR3**, LHCGR, **MYLK3**, NOS2, NOS3, **ORAI2**, OXTR, P2RX1, P2RX7, PDE1A, PDGFRA, PDGFRB, PHKA1, PHKB, PHKG1, PLCB1, PLCB2, **PLCB3**, PLCB4, **PLCD1**, **PLCD3**, **PLCG2**, PLCZ1, **PLN**, **PPIF**, PPP3CA, PPP3CB, PPP3CC, PRKCA, PRKCG, **PTAFR**, PTGFR, PTK2B, RYR1, RYR3, **SLC25A5**, SLC8A1, TACR1, TNNC2, **VDAC1**, **VDAC2**, **VDAC3** | 8.77E-03*<br>1.06E-05***<br>1.33E-02*** |

| | | | |
|---|---|---|---|
| | Ras signalling pathway (fca04014) | ABL1, AKT3, ANGPT4, CSF1R, EGF, EGFR, EXOC2, FGF12, FGF13, FGF7, FGFR1, GAB1, GAB2, GRIN2A, GRIN2B, HGF, IGF1, INSR, KDR, KIT, KRAS, KSR1, KSR2, MAPK10, MAPK8, NF1, NFKB1, PAK5, PAK6, PDGFC, PDGFD, PDGFRA, PDGFRB, PIK3CA, PIK3CG, PIK3R1, PLA2G4D, PLA2G4E, PLA2G5, PRKCA, PRKCG, RAC2, RASA1, RASA2, RASA3, RASAL2, RASAL3, RASGRP3, RASGRP4, RASSF5, REL, RGL1, SOS1, SOS2, TEK, TIAM1, VEGFC, ZAP70 | 1.66E-03*** |
| | AMPK signalling pathway (fca04152) | ACACA, ADIPOQ, ADRA1A, AKT3, CAB39L, CAMKK1, CAMKK2, CD36, CFTR, CREB1, CREB3L1, CREB3L2, CREB5, FBP1, FBP2, GYS2, IGF1, INSR, LEPR, PCK1, PFKFB1, PFKFB4, PIK3CA, PIK3CG, PIK3R1, PPARG, PPP2R2A, PPP2R2B, PPP2R3A, PPP2R5A, PRKAA2, PRKAG3, RHEB, RPTOR | 1.31E-02*** |
| | Chemokine signalling pathway (fca04062) | ADCY2, ADCY4, ADCY5, ADCY8, ADCY9, AKT3, ARRB1, BRAF, CCL14, CCL19, CCL21, CCL8, CCR1, CXCR2, DOCK2, ELMO1, FGR, GRK4, GRK5, GRK7, ITK, JAK2, JAK3, KRAS, NFKB1, PARD3, PIK3CA, PIK3CG, PIK3R1, PLCB1, PLCB2, PLCB4, PTK2, PTK2B, RAC2, ROCK1, SOS1, SOS2, SRC, STAT3, TIAM1, VAV3, WAS | 1.38E-02*** |
| | NF-kappa B signalling pathway (fca04064) | ATM, BTK, CARD11, CCL19, CCL21, CD14, CD40, CFLAR, IL1B, LBP, LCK, LTB, MALT1, MAP3K14, NFKB1, PLAU, PRKCQ, SYK, TLR4, TNF, TNFRSF11A, TNFSF11, TNFSF13B, TRAF5, VCAM1, ZAP70 | 1.05E-02*** 2.56E-02*** |
| | ErbB signalling pathway (fca04012) | ABL1, AKT3, BRAF, CAMK2A, CAMK2B, CAMK2D, CAMK2G, CBL, CBLB, EGF, EGFR, GAB1, KRAS, MAPK10, MAPK8, PAK5, PAK6, PIK3CA, PIK3CG, PIK3R1, PRKCA, PRKCG, PTK2, SOS1, SOS2, SRC | 1.05E-02*** |
| | TNF signalling pathway (fca04668) | AKT3, ATF2, CASP10, CASP8, CFLAR, CREB1, CREB3L1, CREB3L2, CREB5, IL18R1, IL1B, ITCH, MAP2K6, MAP3K14, MAP3K5, MAP3K8, MAPK10, MAPK14, MAPK8, MMP9, NFKB1, NOD2, PIK3CA, PIK3CG, PIK3R1, RIPK3, RPS6KA5, TNF, TRAF5, VCAM1 | 6.88E-03*** |
| | Phosphatidylinositol signalling system (fca04070) | CDS1, DGKG, DGKH, DGKI, ENSFCAG00000027562, INPP4A, INPP5B, INPP5D, ITPKB, ITPR1, ITPR2, MTM1, MTMR7, PIK3C2G, PIK3C3, PIK3CA, PIK3CG, PIK3R1, PIP4K2A, PIP5K1B, PLCB1, PLCB2, PLCB4, PLCZ1, PPIP5K2, PRKCA, PRKCG, PTEN, SYNJ1 | 7.22E-03*** 9.67E-03*** |
| Endocrine-related | Progesterone-mediated oocyte maturation (fca04914) | ADCY2, **ADCY3**, ADCY4, ADCY5, ADCY8, ADCY9, AKT3, ANAPC10, **ANAPC2**, BRAF, **BUB1**, **CCNA2**, **CCNB1**, **CCNB2**, **CDC25A**, **CDC25B**, **CDK1**, **CDK2**, CPEB1, CPEB4, **FZR1**, **HSP90AA1**, **HSP90AB1**, IGF1, KRAS, MAD1L1, **MAD2L1**, MAPK10, **MAPK11**, MAPK14, MAPK8, PIK3CA, PIK3CG, PIK3R1, **PKMYT1**, **PLK1**, RPS6KA3, RPS6KA6 | 4.95E-02* |
| | Thyroid hormone synthesis (fca04918) | ADCY2, **ADCY3**, ADCY4, ADCY5, ADCY8, ADCY9, ATF2, **ATF4**, **ATP1A1**, ATP1A4, **ATP1B3**, **CANX**, CREB1, CREB3L1, CREB3L2, CREB5, DUOXA2, GNA14, GPX3, GPX5, GPX6, **GPX8**, **HSP90B1**, **HSPA5**, ITPR1, ITPR2, **ITPR3**, IYD, LRP2, **PDIA4**, PLCB1, PLCB2, **PLCB3**, PLCB4, PRKCA, PRKCG, **TSHR** | 4.01E-03* 1.05E-03*** |
| | Glucagon signalling pathway (fca04922) | ACACA, ACACB, ADCY2, AKT3, ATF2, **ATF4**, CAMK2A, CAMK2B, CAMK2D, CAMK2G, **CPT1B**, CREB1, CREB3L1, CREB3L2, CREB5, **CRTC2**, **G6PC3**, GNA14, **GYS1**, GYS2, ITPR1, ITPR2, **ITPR3**, **LDHA**, PCK1, **PCK2**, **PDHA1**, | 4.01E-03* 1.05E-04*** 4.46E-02*** |

| | | | |
|---|---|---|---|
| | | *PFKFB1, PGAM2, PHKA1, PHKB, PHKG1, **PKM**, PLCB1, PLCB2, **PLCB3**, PLCB4, PPARA, PPP3CA, PPP3CB, PPP3CC, **PPP4C**, PRKAA2, PRKAG3, PYGM, SIK2, **SLC2A1*** | |
| | GnRH signalling pathway (fca04912) | *ADCY2, **ADCY3**, ADCY4, ADCY5, ADCY8, ADCY9, **ATF4**, CACNA1C, CACNA1D, CAMK2A, CAMK2B, CAMK2D, CAMK2G, EGFR, GNA14, GNRHR, **HRAS**, ITPR1, ITPR2, **ITPR3**, KRAS, MAP2K6, MAP3K1, MAP3K3, MAPK10, **MAPK11**, MAPK14, MAPK7, MAPK8, **MMP14**, MMP2, PLA2G4D, PLA2G4E, PLCB1, PLCB2, **PLCB3**, PLCB4, PTK2B, SOS1, SOS2, SRC* | 8.77E-03*<br>1.06E-05***<br>1.21E-02*** |
| | Oxytocin signalling pathway (fca04921) | *ADCY2, **ADCY3**, ADCY4, ADCY5, ADCY8, ADCY9, CACNA1C, CACNA1D, CACNA2D1, CACNA2D2, CACNA2D3, **CACNA2D4**, **CACNB3**, CACNB4, CACNG2, CACNG7, CAMK1G, CAMK2A, CAMK2B, CAMK2D, CAMK2G, CAMK4, CAMKK1, CAMKK2, **CDKN1A**, **EEF2**, EGFR, GNA14, GUCY1A1, GUCY1B1, **HRAS**, ITPR1, ITPR2, **ITPR3**, **KCNJ4**, KCNJ6, KRAS, MAPK7, MEF2C, **MYL6**, **MYL6B**, **MYLK3**, NFATC3, NOS3, OXTR, PLA2G4D, PLA2G4E, PLCB1, PLCB2, **PLCB3**, PLCB4, PPP3CA, PPP3CB, PPP3CC, PRKAA2, PRKAG3, PRKCA, PRKCG, RGS2, ROCK1, RYR1, RYR3, SRC, TRPM2* | 8.77E-03*<br>3.59E-06***<br>9.67E-03*** |
| | Aldosterone synthesis and secretion (fca04925) | *ADCY2, **ADCY3**, ADCY4, ADCY5, ADCY8, ADCY9, AGTR1, ATF2, **ATF4**, CACNA1C, CACNA1D, CAMK1G, CAMK2A, CAMK2B, CAMK2D, CAMK2G, CAMK4, CREB1, CREB3L1, CREB3L2, CREB5, CYP21A2, GNA14, HSD3B2, ITPR1, ITPR2, **ITPR3**, **LDLR**, PDE2A, PLCB1, PLCB2, **PLCB3**, PLCB4, PRKCA, PRKCE, PRKCG, PRKD1* | 1.61E-02*<br>1.42E-05***<br>1.95E-02*** |
| | Oestrogen signalling pathway (fca04915) | *ADCY2, **ADCY3**, ADCY4, ADCY5, ADCY8, ADCY9, AKT3, ATF2, **ATF4**, CREB1, CREB3L1, CREB3L2, CREB5, EGFR, ESR1, **FKBP4**, FKBP5, GABBR2, GNA14, **HRAS**, **HSP90AA1**, **HSP90AB1**, **HSP90B1**, **HSPA2**, **HSPA8**, ITPR1, ITPR2, **ITPR3**, KCNJ6, KRAS, MMP2, MMP9, NOS3, PIK3CA, PIK3CG, PIK3R1, PLCB1, PLCB2, **PLCB3**, PLCB4, SOS1, SOS2, SRC* | 2.22E-02*<br>6.36E-04*** |
| | Insulin secretion (fca04911) | *ADCY2, ADCY4, ADCY5, ADCY8, ADCY9, ADCYAP1R1, ATF2, ATP1A4, CACNA1C, CACNA1D, CAMK2A, CAMK2B, CAMK2D, CAMK2G, CREB1, CREB3L1, CREB3L2, CREB5, GNA14, KCNMB4, PCLO, PLCB1, PLCB2, PLCB4, PRKCA, PRKCG, RAPGEF4, SNAP25, STX1A* | 8.19E-04*** |
| | Insulin resistance (fca00510) | *ACACB, AKT3, CD36, CREB1, CREB3L1, CREB3L2, CREB5, GFPT2, GYS2, INSR, MAPK10, MAPK8, MLXIPL, NFKB1, NOS3, NR1H3, OGA, PCK1, PIK3CA, PIK3CG, PIK3R1, PPARA, PPP1R3A, PRKAA2, PRKAG3, PRKCE, PRKCQ, PTEN, PTPN1, PYGM, RPS6KA3, RPS6KA6, SLC27A2, SLC27A6, STAT3, TNF* | 4.00E-04*** |
| | Renin secretion (fca04924) | *ADCY5, ADCYAP1R1, AGTR1, AQP1, CACNA1C, CACNA1D, CLCA1, CLCA2, CLCA4, CREB1, ENSFCAG00000028907, GNA14, GUCY1A1, GUCY1B1, ITPR1, ITPR2, PDE1A, PLCB1, PLCB2, PLCB4, PPP3CA, PPP3CB, PPP3CC* | 1.57E-03***<br>1.74E-02*** |
| | Prolactin signalling pathway (fca04917) | *AKT3, CISH, ELF5, ESR1, JAK2, KRAS, LHCGR, MAPK10, MAPK14, MAPK8, NFKB1, PIK3CA, PIK3CG, PIK3R1, PRL, PRLR, SOCS2, SOS1, SOS2, SRC, STAT3, TNFRSF11A, TNFSF11* | 6.88E-03*** |

| | | | |
|---|---|---|---|
| Immune-related | Platelet activation (fca04611) | ADCY2, **ADCY3**, ADCY4, ADCY5, ADCY8, ADCY9, AKT3, APBB1IP, **ARHGAP35**, ARHGEF12, BTK, **COL11A2**, COL1A1, COL1A2, **COL2A1**, COL3A1, ENSFCAG00000029640, FERMT3, FGG, **GNA13**, GNA14, GUCY1A1, GUCY1B1, ITGA2B, **ITGB1**, ITGB3, ITPR1, ITPR2, **ITPR3**, LCP2, **MAPK11**, MAPK14, **MYL12A**, **MYLK3**, NOS3, P2RX1, **P2RY1**, PIK3CA, PIK3CG, PIK3R1, PLA2G4D, PLA2G4E, PLCB1, PLCB2, **PLCB3**, PLCB4, **PLCG2**, PTGS1, **RAP1B**, ROCK1, SRC, SYK, **TLN1**, **VAMP8**, **VASP**, VWF | 9.66E-03*<br>5.78E-04***<br>1.95E-02*** |
| | T cell receptor signalling pathway (fca04660) | AKT3, CARD11, CD28, CD3D, CD3E, CTLA4, DLG1, GRAP2, IL4, ITK, KRAS, LCK, LCP2, MALT1, MAP3K14, MAP3K8, MAPK14, NFATC3, NFKB1, PAK5, PAK6, PIK3CA, PIK3CG, PIK3R1, PPP3CA, PPP3CB, PPP3CC, PRKCQ, PTPN6, PTPRC, SOS1, SOS2, TEC, TNF, VAV3, ZAP70 | 9.74E-05***<br>9.67E-03*** |
| | B cell receptor signalling pathway (fca04662) | AKT3, BTK, CARD11, CR2, DAPP1, INPP5D, KRAS, MALT1, NFATC3, NFKB1, PIK3AP1, PIK3CA, PIK3CG, PIK3R1, PPP3CA, PPP3CB, PPP3CC, PTPN6, RAC2, RASGRP3, SOS1, SOS2, SYK, VAV3 | 2.71E-03***<br>3.22E-02*** |
| | Complement and coagulation cascades (fca04610) | A2M, C1QA, C1QB, C1S, C3AR1, C4BPB, C5, C7, C8A, C8B, C9, CR2, ENSFCAG00000000177, ENSFCAG00000013840, F13A1, F3, F5, FGG, KNG1, MASP1, PLAU, PLG, SERPIND1, SERPING1, TFPI, VWF | 1.28E-03*** |
| | Fc epsilon RI signalling pathway (fca04664) | AKT3, BTK, FCER1A, GAB2, IL4, INPP5D, KRAS, LCP2, MAP2K6, MAPK10, MAPK14, MAPK8, PIK3CA, PIK3CG, PIK3R1, PLA2G4D, PLA2G4E, RAC2, SOS1, SOS2, SYK, TNF, VAV3 | 2.73E-03*** |
| | Hematopoietic cell lineage (fca04640) | CD14, CD2, CD34, CD36, CD3D, CD3E, CD5, CR2, CSF1R, FCGR3, IL1B, IL2RA, IL4, IL4R, IL5RA, IL6R, IL7R, ITGA1, ITGA2B, ITGA4, ITGA5, ITGA6, ITGAM, ITGB3, KIT, MME, TNF | 4.12E-03***<br>1.33E-02*** |
| | Primary immunodeficiency (fca05340) | ADA, BTK, CD3D, CD3E, CD40, CIITA, DCLRE1C, LCK, TNFRSF13B, ZAP70 | 4.46E-02*** |
| | Fc gamma R-mediated phagocytosis (fca04666) | AKT3, ASAP1, ASAP3, DOCK2, FCGR2B, FCGR3, GAB2, INPP5D, LIMK2, PIK3CA, PIK3CG, PIK3R1, PIP5K1B, PLPP1, PLPP3 , PRKCA, PRKCE, PRKCG, PTPRC, RAC2, SCIN, SYK, VAV3, WAS, WASF2 | 1.10E-02*** |
| | Leukocyte transendothelial migration (fca04670) | ACTN2, ACTN3, CDH5, CLDN4, CLDN7, CLDN8, ITGA4, ITGAM, ITGB2, ITK, JAM2, MAPK14, MMP2, MMP9, NCF4, OCLN, PECAM1, PIK3CA, PIK3CG, PIK3R1, PRKCA, PRKCG, PTK2, PTK2B, RAC2, RAPGEF3, RAPGEF4, RASSF5, ROCK1, TXK, VAV3, VCAM1, VCL | 6.57E-03*** |
| Neurologic system-related | Dopaminergic synapse (fca04728) | ADCY5, AKT3, ATF2, **ATF4**, CACNA1A, CACNA1C, CACNA1D, CAMK2A, CAMK2B, CAMK2D, CAMK2G, CLOCK, **COMT**, CREB1, CREB3L1, CREB3L2, CREB5, DRD3, GNA14, GRIA1, GRIA3, GRIA4, GRIN2A, GRIN2B, **GSK3A**, ITPR1, ITPR2, **ITPR3**, KCNJ6, **KIF5B**, MAOA, MAOB, MAPK10, **MAPK11**, MAPK14, MAPK8, PLCB1, PLCB2, **PLCB3**, PLCB4, PPP1R1B, **PPP2R1A**, PPP2R2A, PPP2R2B, PPP2R3A, PPP2R5A, **PPP2R5D**, PPP3CA, PPP3CB, PPP3CC, PRKCA, PRKCG, SCN1A, SLC18A2 | 1.76E-02*<br>7.65E-06***<br>9.91E-03*** |

| | | | |
|---|---|---|---|
| | Long-term potentiation (fca04720) | *ADCY8*, ***ATF4***, *BRAF*, *CACNA1C*, *CAMK2A*, *CAMK2B*, *CAMK2D*, *CAMK2G*, *CAMK4*, *GNA14*, *GRIA1*, *GRIN2A*, *GRIN2B*, ***HRAS***, *ITPR1*, *ITPR2*, ***ITPR3***, *KRAS*, *PLCB1*, *PLCB2*, ***PLCB3***, *PLCB4*, *PPP1R1A*, *PPP3CA*, *PPP3CB*, *PPP3CC*, *PRKCA*, *PRKCG*, ***RAP1B***, *RAPGEF3*, *RPS6KA3*, *RPS6KA6* | 1.26E-02*<br>4.94E-05***<br>1.21E-02*** |
| | Axon guidance (fca04360) | *ABL1*, *ABLIM1*, *ABLIM3*, *ARHGEF12*, *DCC*, *DPYSL2*, *EPHA1*, *EPHA3*, *EPHA4*, *EPHA7*, *EPHB1*, *FES*, *KRAS*, *LIMK2*, *LRRC4*, *NFATC3*, *NRP1*, *PAK5*, *PAK6*, *PLXNA4*, *PPP3CA*, *PPP3CB*, *PPP3CC*, *PTK2*, *RAC2*, *RASA1*, *ROCK1*, *SEMA3A*, *SEMA3D*, *SEMA3E*, *SEMA3F*, *SEMA3G*, *SEMA4G*, *SEMA5A*, *SEMA6D*, *SLIT1*, *SRGAP1*, *SRGAP2*, *UNC5C*, *UNC5D* | 4.00E-04***<br>1.33E-02*** |
| | Glutamatergic synapse (fca04724) | *ADCY2*, *ADCY4*, *ADCY5*, *ADCY8*, *ADCY9*, *CACNA1A*, *CACNA1C*, *CACNA1D*, *GNA14*, *GRIA1*, *GRIA3*, *GRIA4*, *GRIK1*, *GRIN2A*, *GRIN2B*, *GRIN3A*, *GRM4*, *GRM8*, *HOMER1*, *ITPR1*, *ITPR2*, *PLA2G4D*, *PLA2G4E*, *PLCB1*, *PLCB2*, *PLCB4*, *PPP3CA*, *PPP3CB*, *PPP3CC*, *PRKCA*, *PRKCG*, *SLC1A2*, *SLC1A3*, *SLC1A7*, *SLC38A1* | 8.08E-04*** |
| | Retrograde endocannabinoid signalling (fca04723) | *ABHD6*, *ADCY2*, *ADCY4*, *ADCY5*, *ADCY8*, *ADCY9*, *CACNA1A*, *CACNA1C*, *CACNA1D*, *FAAH*, *GABRA3*, *GABRA4*, *GABRB2*, *GABRR3*, *GNA14*, *GRIA1*, *GRIA3*, *GRIA4*, *ITPR1*, *ITPR2*, *KCNJ6*, *MAPK10*, *MAPK14*, *MAPK8*, *MGLL*, *NAPEPLD*, *PLCB1*, *PLCB2*, *PLCB4*, *PRKCA*, *PRKCG* | 1.98E-03***<br>2.10E-02*** |
| | Serotonergic synapse (fca04726) | *ADCY5*, *ALOX5*, *BRAF*, *CACNA1A*, *CACNA1C*, *CACNA1D*, *CYP4X1*, *GABRB2*, *GNA14*, *HTR2B*, *ITPR1*, *ITPR2*, *KCNJ6*, *KRAS*, *MAOA*, *MAOB*, *PLA2G4D*, *PLA2G4E*, *PLCB1*, *PLCB2*, *PLCB4*, *PRKCA*, *PRKCG*, *PTGS1*, *RAPGEF3*, *SLC18A2*, *SLC6A4*, *TPH1* | 2.42E-02*** |
| | Neurotrophin signalling pathway (fca04722) | *ABL1*, *AKT3*, *BRAF*, *CAMK2A*, *CAMK2B*, *CAMK2D*, *CAMK2G*, *CAMK4*, *GAB1*, *IRAK2*, *IRAK3*, *KRAS*, *MAP3K1*, *MAP3K3*, *MAP3K5*, *MAPK10*, *MAPK14*, *MAPK7*, *MAPK8*, *NFKB1*, *NTF3*, *NTF4*, *NTRK2*, *NTRK3*, *PIK3CA*, *PIK3CG*, *PIK3R1*, *RPS6KA3*, *RPS6KA5*, *RPS6KA6*, *SOS1*, *SOS2* | 1.72E-02*** |
| | Cholinergic synapse (fca04725) | *ADCY2*, *ADCY4*, *ADCY5*, *ADCY8*, *ADCY9*, *AKT3*, *CACNA1A*, *CACNA1C*, *CACNA1D*, *CAMK2A*, *CAMK2B*, *CAMK2D*, *CAMK2G*, *CAMK4*, *CREB1*, *CREB3L1*, *CREB3L2*, *CREB5*, *GNA14*, *ITPR1*, *ITPR2*, *JAK2*, *KCNJ6*, *KRAS*, *PIK3CA*, *PIK3CG*, *PIK3R1*, *PLCB1*, *PLCB2*, *PLCB4*, *PRKCA*, *PRKCG* | 4.64E-03*** |
| Cancer-related | Choline metabolism in cancer (fca05231) | *AKT3*, ***CHKB***, *DGKG*, *DGKH*, *DGKI*, ***DGKQ***, ***DGKZ***, *EGF*, *EGFR*, ***EIF4EBP1***, *GPCPD1*, ***HIF1A***, ***HRAS***, *KRAS*, ***LYPLA1***, *MAPK10*, *MAPK8*, ***PDGFB***, *PDGFC*, *PDGFD*, *PDGFRA*, *PDGFRB*, *PIK3CA*, *PIK3CG*, *PIK3R1*, *PIP5K1B*, *PLA2G4D*, *PLA2G4E*, *PLPP1*, *PLPP3*, *PRKCA*, *PRKCG*, *RAC2*, *RHEB*, *SLC22A1*, *SLC22A2*, *SLC22A3*, *SLC44A1*, *SLC44A3*, *SLC44A5*, *SOS1*, *SOS2*, *WAS*, *WASF2* | 4.73E-02*<br>1.05E-04***<br>2.86E-02*** |
| | MicroRNAs in cancer (fca05206) | *ABCB1*, *ABL1*, *APC*, *ATM*, *BMPR2*, ***BRCA1***, ***CDC25A***, ***CDC25B***, ***CDCA5***, ***CDKN1A***, ***CDKN2A***, ***DDIT4***, ***DNMT1***, *DNMT3A*, ***E2F2***, *EGFR*, ***ERBB3***, ***EZR***, ***GLS2***, ***HRAS***, *ITGA5*, *ITGB3*, ***KIF23***, *KRAS*, *MAPK7*, ***MDM2***, *MDM4*, *MMP16*, *MMP9*, *NFKB1*, ***NOTCH1***, *NOTCH3*, ***PAK4***, *PDCD4*, ***PDGFB***, *PDGFRA*, *PDGFRB*, *PLAU*, ***PLCG2***, *PRKCA*, *PRKCE*, *PRKCG*, *PTEN*, ***RDX***, *RECK*, *ROCK1*, *RPS6KA5*, | 8.77E-03*<br>1.52E-02*** |

| | | | |
|---|---|---|---|
| | | RPTOR, **SERPINB5**, **SLC7A1**, SOS1, SOS2, ST14, STAT3, **THBS1**, TNC, **TP53**, TP63, **UBE2I**, **VEGFA**, ZEB1, ZEB2 | |
| | Prostate cancer (fca05215) | AKT3, AR, BRAF, CREB1, CREB3L1, CREB3L2, CREB5, EGF, EGFR, FGFR1, IGF1, KRAS, NFKB1, PDGFC, PDGFD, PDGFRA, PDGFRB, PIK3CA, PIK3CG, PIK3R1, PTEN, RB1, SOS1, SOS2, TCF7, TCF7L1 | 1.18E-02*** |
| | Glioma (fca05214) | AKT3, BRAF, CAMK2A, CAMK2B, CAMK2D, CAMK2G, EGF, EGFR, IGF1, KRAS, PDGFRA, PDGFRB, PIK3CA, PIK3CG, PIK3R1, PRKCA, PRKCG, PTEN, RB1, SOS1, SOS2 | 1.20E-02*** |
| | Pathways in cancer (fca05200) | ABL1, ADCY2, ADCY4, ADCY5, ADCY8, ADCY9, AGTR1, AKT3, APC, AR, ARHGEF12, ARNT, BCR, BRAF, BRCA2, CASP8, CBL, CBLB, COL4A4, COL4A6, CSF1R, CTBP2, DAPK1, DCC, EDNRB, EGF, EGFR, FGF12, FGF13, FGF7, FGFR1, GLI3, GNA14, HGF, HHIP, IGF1, ITGA2B, ITGA6, JAK1, KIT, KRAS, LAMA2, LAMA3, LAMB4, LAMC2, LAMC3, LPAR1, LPAR6, MAPK10, MAPK8, MECOM, MITF, MLH1, MMP2, MMP9, MSH3, NFKB1, NOS2, PDGFRA, PDGFRB, PIK3CA, PIK3CG, PIK3R1, PLCB1, PLCB2, PLCB4, PPARG, PRKCA, PRKCG, PTCH1, PTCH2, PTEN, PTK2, RAC2, RARB, RASGRP3, RASGRP4, RASSF5, RB1, ROCK1, RUNX1T1, RXRG, SMAD2, SOS1, SOS2, SPI1, STAT3, TCF7, TCF7L1, TGFBR2, TRAF5, VEGFC | 1.66E-03*** |
| | Proteoglycans in cancer (fca05205) | AKT3, ANK2, ARHGEF12, BRAF, CAMK2A, CAMK2B, CAMK2D, CAMK2G, CBL, CBLB, DCN, DROSHA, EGFR, ESR1, FGFR1, FLNC, GAB1, GPC3, HCLS1, HGF, IGF1, ITGA5, ITGB3, ITPR1, ITPR2, KDR, KRAS, LUM, MAPK14, MMP2, MMP9, NANOG, PDCD4, PIK3CA, PIK3CG, PIK3R1, PLAU, PRKCA, PRKCG, PTCH1, PTK2, PTPN6, ROCK1, SOS1, SOS2, SRC, STAT3, TIAM1, TLR4, TNF, VTN | 4.31E-03*** |
| Sensory system | Inflammatory mediator regulation of TRP channels (fca04750) | ADCY2, **ADCY3**, ADCY4, ADCY5, ADCY8, ADCY9, CAMK2A, CAMK2B, CAMK2D, CAMK2G, **CYP2J2**, GNA14, HTR2B, IGF1, IL1B, ITPR1, ITPR2, **ITPR3**, MAP2K6, MAPK10, **MAPK11**, MAPK14, MAPK8, **NTRK1**, PIK3CA, PIK3CG, PIK3R1, PLA2G4D, PLA2G4E, PLCB1, PLCB2, **PLCB3**, PLCB4, **PLCG2**, PRKCA, PRKCE, PRKCG, PRKCH, PRKCQ, SRC, TRPA1, TRPV1, **TRPV2**, **TRPV4** | 8.77E-03*<br>3.05E-05***<br>9.91E-03*** |
| Membrane transport | ABC transporters (fca02010) | ABCA1, ABCA12, ABCA6, ABCA8, ABCA9, ABCB1, ABCB11, ABCB5, ABCB7, ABCC11, ABCC2, ABCC4, ABCC5, ABCC9, ABCD3, ABCG2, ABCG5, ABCG8, CFTR | 6.36E-04*** |
| Development and regeneration | Osteoclast differentiation (fca04380) | AKT3, BTK, CAMK4, CREB1, CSF1R, CYBB, FCGR2B, FCGR3, FCGR3A, FOSB, GAB2, IL1B, ITGB3, JAK1, LCK, LCP2, MAP2K6, MAP3K14, MAPK10, MAPK14, MAPK8, MITF, NCF4, NFKB1, PIK3CA, PIK3CG, PIK3R1, PPARG, PPP3CA, PPP3CB, PPP3CC, SPI1, SYK, TEC, TGFBR2, TNF, TNFRSF11A, TNFSF11, TREM2, TYROBP | 2.08E-04*** |
| Environmental adaptation | Circadian entrainment (fca04713) | ADCY10, ADCY2, ADCY4, ADCY5, ADCY8, ADCY9, ADCYAP1R1, CACNA1C, CACNA1D, CAMK2A, CAMK2B, CAMK2D, CAMK2G, CREB1, GNA14, GRIA1, GRIA3, GRIA4, GRIN2A, GRIN2B, GUCY1A1, GUCY1B1, ITPR1, KCNJ6, PER1, PLCB1, PLCB2, PLCB4, PRKCA, PRKCG, RPS6KA5, RYR1, RYR3 | 2.52E-04***<br>4.46E-02*** |
| | Circadian rhythm (fca04710) | BTRC, CLOCK, CREB1, CRY1, CRY2, CUL1, PER1, PRKAA2, PRKAG3, RORA, RORB, RORC | 2.02E-02*** |

KEGG, Kyoto Encyclopedia of Genes and Genomes; and DEGs, differentially expressed genes. Gene symbols not available replaced with ensembl ID. DEGs in analysis: 6210, up-regulated genes (n = 2554), down-regulated genes (n = 3656), exclusively up-regulated genes (n = 1442), and exclusively down-regulated genes (n = 2426). Up-regulated DEGs in bold, down-regulated DEGs in non-bold, exclusive DEGs are underlined. *FDR for total DEGs (up- and down-regulated together), **FDR for up-regulated DEGs, and ***FDR for down-regulated DEGs, ****FDR for exclusively up/ and down/regulated DEGs together. FDR for exclusive DEGs (up- or downregulated) are underlined. Data based on average of logarithmic scale base two ($log_2$). Data analysed with DAVID (https://david.ncifcrf.gov/).

**Supplementary table 10.** Collagen molecules expression across sample groups.

| Gene symbol | Ensemble ID | Cell line | LB *f*HER2-tumour | Normal-like TN tumour | Basal-like TN tumour |
|---|---|---|---|---|---|
| *COL11A2* | ENSFCAG00000004005 | 1 | 0 | 1 | 0 |
| *COL14A1* | ENSFCAG00000001462 | -1 | 0 | -1 | -1 |
| *COL16A1* | ENSFCAG00000015669 | 0 | 1 | 0 | 0 |
| *COL1A1* | ENSFCAG00000004564 | -1 | 1 | 0 | 1 |
| *COL1A2* | ENSFCAG00000030892 | -1 | 1 | 0 | 1 |
| *COL20A1* | ENSFCAG00000008920 | 1 | 0 | 0 | 0 |
| *COL21A1* | ENSFCAG00000029537 | -1 | -1 | 0 | -1 |
| *COL26A1* | ENSFCAG00000008073 | 0 | 0 | 1 | 1 |
| *COL28A1* | ENSFCAG00000018957 | -1 | 0 | 0 | -1 |
| *COL2A1* | ENSFCAG00000014002 | 1 | 1 | 1 | 1 |
| *COL3A1* | ENSFCAG00000014740 | -1 | 1 | 0 | 0 |
| *COL4A1* | ENSFCAG00000014784 | 1 | 1 | 0 | 0 |
| *COL4A2* | ENSFCAG00000014785 | 0 | 1 | 0 | 0 |
| *COL4A4* | ENSFCAG00000008677 | -1 | 0 | 0 | 0 |
| *COL4A5* | ENSFCAG00000000758 | 1 | 0 | 0 | 0 |
| *COL4A6* | ENSFCAG00000005189 | -1 | 0 | 0 | 0 |
| *COL5A2* | ENSFCAG00000014741 | 0 | 1 | 0 | 0 |
| *COL5A3* | ENSFCAG00000009383 | 0 | 0 | -1 | -1 |
| *COL6A1* | ENSFCAG00000014813 | -1 | 0 | 0 | 0 |
| *COL6A2* | ENSFCAG00000014814 | 0 | 1 | 0 | 0 |
| *COL6A3* | ENSFCAG00000012720 | -1 | 1 | 0 | 0 |
| *COL6A6* | ENSFCAG00000013504 | -1 | 0 | 0 | 0 |
| *COL8A1* | ENSFCAG00000011935 | -1 | 0 | 0 | 0 |
| *COL9A1* | ENSFCAG00000010489 | -1 | 0 | 0 | 0 |
| *COL9A3* | ENSFCAG00000001332 | 1 | 1 | 1 | 1 |
| *COLEC12* | ENSFCAG00000015762 | -1 | 0 | -1 | 0 |
| *COLGALT2* | ENSFCAG00000012642 | 1 | 0 | 1 | 0 |
| *COLQ* | ENSFCAG00000011304 | -1 | 0 | 0 | 0 |

0 = not dysregulated (gene log2 fold change relative to controls > -2 and < 2), -1 = downregulated (gene log2 fold change relative to controls ≤ -2), and 1 = upregulated (gene log2 fold change relative to controls ≥ 2). LB; Luminal B, and TN; triple-negative. Data based on average of logarithmic scale base two ($log_2$).

## References


1. McNeill, C.J., et al., *Evaluation of adjuvant doxorubicin-based chemotherapy for the treatment of feline mammary carcinoma.* J Vet Intern Med, 2009. **23**(1): p. 123-9.
2. Mohsin, S.K., et al., *Progesterone receptor by immunohistochemistry and clinical outcome in breast cancer: a validation study.* Mod Pathol, 2004. **17**(12): p. 1545-54.
3. Soares, M., et al., *Immunophenotyping of primary and metastatic lesions in feline mammary tumors - are they equal?* Microscopy and Microanalysis, 2013. **19**(S4): p. 19-20.
4. Soares, M., et al., *Molecular based subtyping of feline mammary carcinomas and clinicopathological characterization.* Breast, 2016. **27**: p. 44-51.
5. Park, S., et al., *Characteristics and outcomes according to molecular subtypes of breast cancer as classified by a panel of four biomarkers using immunohistochemistry.* Breast, 2012. **21**(1): p. 50-7.
6. Soares, M., et al., *Feline HER2 protein expression levels and gene status in feline mammary carcinoma: optimization of immunohistochemistry (IHC) and in situ hybridization (ISH) techniques.* Microsc Microanal, 2013. **19**(4): p. 876-82.
7. Wolff, A.C., et al., *Human Epidermal Growth Factor Receptor 2 Testing in Breast Cancer: American Society of Clinical Oncology/College of American Pathologists Clinical Practice Guideline Focused Update.* Arch Pathol Lab Med, 2018. **142**(11): p. 1364-1382.
8. Granados-Soler, J.L., et al., *Analysis of Copy-Number Variations and Feline Mammary Carcinoma Survival.* Sci Rep, 2020. **10**(1): p. 1003.
9. Brunetti, B., et al., *Molecular phenotype in mammary tumours of queens: correlation between primary tumour and lymph node metastasis.* J Comp Pathol, 2013. **148**(2-3): p. 206-13.
10. Soares, M., et al., *Ki-67 as a Prognostic Factor in Feline Mammary Carcinoma: What Is the Optimal Cutoff Value?* Vet Pathol, 2016. **53**(1): p. 37-43.
11. Elston, C.W. and I.O. Ellis, *Pathological prognostic factors in breast cancer. I. The value of histological grade in breast cancer: experience from a large study with long-term follow-up.* Histopathology, 1991. **19**(5): p. 403-10.
12. Castagnaro, M., et al., *Tumour grading and the one-year post-surgical prognosis in feline mammary carcinomas.* J Comp Pathol, 1998. **119**(3): p. 263-75.
13. Mills, S.W., et al., *Prognostic value of histologic grading for feline mammary carcinoma: a retrospective survival analysis.* Vet Pathol, 2015. **52**(2): p. 238-49.
14. Dagher, E., et al., *Feline Invasive Mammary Carcinomas: Prognostic Value of Histological Grading.* Vet Pathol, 2019. **56**(5): p. 660-670.
